# Carbon Nano-onions: Potassium Intercalation and Reductive Covalent Functionalization

**DOI:** 10.1021/jacs.1c07604

**Published:** 2021-10-26

**Authors:** M. Eugenia Pérez-Ojeda, Edison Castro, Claudia Kröckel, Matteo Andrea Lucherelli, Ursula Ludacka, Jani Kotakoski, Katharina Werbach, Herwig Peterlik, Manuel Melle-Franco, Julio C. Chacón-Torres, Frank Hauke, Luis Echegoyen, Andreas Hirsch, Gonzalo Abellán

**Affiliations:** † Department of Chemistry and Pharmacy, Chair of Organic Chamistry II, Friedrich-Alexander University of Erlangen-Nuremberg, Nikolaus-Fiebiger-Str. 10, 91058 Erlangen, Germany; ‡ Joint Institute of Advanced Materials and Processes (ZMP), Friedrich-Alexander University of Erlangen-Nuremberg, Dr.-Mack-Str. 81, D-90762 Fürth, Germany; § Department of Chemistry, University of Texas at El Paso, El Paso, Texas 79968, United States; ∥ Instituto de Ciencia Molecular, Universidad de Valencia, Catedrático José Beltrán 2, 46980 Paterna, Spain; ⊥ Faculty of Physics, University of Vienna, Boltzmanngasse 5, 1090 Vienna, Austria; # CICECO-Aveiro Institute of Materials, Department of Chemistry, University of Aveiro, 3810-193, Aveiro, Portugal; ∇ School of Physical Sciences and Nanotechnology, Yachay Tech University, 100119-Urcuquí, Ecuador

## Abstract

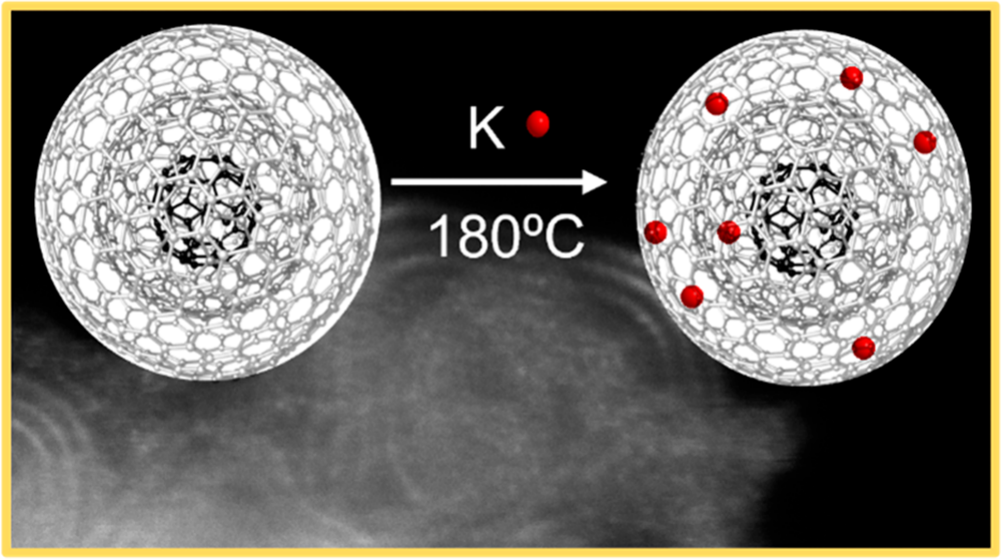

Herein we report the synthesis of covalently functionalized carbon nano-onions (CNOs) via a reductive approach using unprecedented alkali-metal CNO intercalation compounds. For the first time, an *in situ* Raman study of the controlled intercalation process with potassium has been carried out revealing a Fano resonance in highly doped CNOs. The intercalation was further confirmed by electron energy loss spectroscopy and X-ray diffraction. Moreover, the experimental results have been rationalized with DFT calculations. Covalently functionalized CNO derivatives were synthesized by using phenyl iodide and *n*-hexyl iodide as electrophiles in model nucleophilic substitution reactions. The functionalized CNOs were exhaustively characterized by statistical Raman spectroscopy, thermogravimetric analysis coupled with gas chromatography and mass spectrometry, dynamic light scattering, UV–vis, and ATR-FTIR spectroscopies. This work provides important insights into the understanding of the basic principles of reductive CNOs functionalization and will pave the way for the use of CNOs in a wide range of potential applications, such as energy storage, photovoltaics, or molecular electronics.

## Introduction

Since the discovery of the fullerene C_60_ in 1985,^1^ carbon nanomaterials have been investigated in different fields such as photovoltaics and biological applications.^2,3^ In 1992, Ugarte^4^ reported a new allotropic form of carbon known as carbon nano-onions (CNOs), which were first observed by Ijima in 1980.^5^ These multishell fullerenes have received considerable attention during the past decade due to their outstanding properties such as a large surface area to volume ratio, low densities, and a graphitic multilayer morphology, which makes them excellent candidates for diverse applications, such as energy conversion and storage,^6^ supercapacitors,^7^ solid lubricants,^8,9^ and cellular imaging and theranostics,^10,11^ among others.^4,12−15^

CNOs can be synthesized through several methods, such as ball milling,^16^ ion implantation,^17^ arc discharge,^18^ plasma treatment,^19^ chemical vapor deposition,^20^ laser ablation,^21^ electron irradiation,^22^ thermal decomposition of layered nanoreactors,^23,24^ soft-chemistry methods,^25^ and thermal annealing of detonation nanodiamonds (d-NDs) under either vacuum^26^ or inert atmosphere.^27,28^ The most common method for the synthesis of CNOs is based on the thermal annealing of d-NDs of about 5 nm diameter under a He atmosphere at temperatures between 1500 and 1800 °C.^29^ Moreover, this method is highly reproducible and CNOs are obtained in quantitative yields, thus allowing a great potential for industrial applications.

Chemical functionalization has been extensively explored in order to modify the interfacial properties and modulate the solubility of CNOs, representing the most promising route for controlling their processability.^11,12,29,30^ Their degree of functionalization is commonly associated with the size and strain of the CNOs. Typically, small-size CNOs (5–10 nm) are more reactive than larger-sized ones due to their higher curvature, leading to an increase in the pyramidalization of the C atoms.^28^ Different synthetic procedures for CNO functionalization have been investigated using always neutral CNOs; however, the unambiguous characterization of the covalent functionalization in CNOs remains challenging. When it comes to other carbon allotropes, one of the probably most efficient routes for functionalization is the reduction using alkali metals as performed in graphite intercalation compounds (GICs), followed by the quenching of the intermediately formed *graphenides*(2,31) with electrophiles.^3,32,33^ Concerning the application of reductive approaches to CNOs, the number of examples is very scarce, with only one precedent employing a liquid Na–K alloy to activate the surface of the CNOs prior to the reaction with C_16_Br.^30^ It is important to highlight that CNOs exhibit great potential as electrode materials in energy storage devices in which thin-film processing and alkali-metal intercalation are key aspects; therefore, understanding these processes is a matter of utmost importance. Moreover, a controlled reductive functionalization would allow one to envision novel hybrid materials, including covalent intercarbon-allotrope architectures. However, the solid-state intercalation of CNOs with potassium and its ulterior functionalization with electrophiles remains completely unexplored.

Herein, we developed the vapor-phase intercalation of CNOs with alkali metals using high-vacuum conditions as well as a bulk solid-state reaction. For the first time, an *in situ* Raman study of the controlled intercalation process with potassium has been carried out in CNOs, revealing the development of a Fano resonance. Moreover, X-ray diffraction (XRD) and transmission electron microscopy (TEM) analysis allowed a direct observation of the change in the interlayer distance in CNOs after their intercalation and the localization of potassium atoms, respectively. Last but not least, we analyzed the functionalized CNOs by statistical Raman spectroscopy^34,35^ (SRS) and thermogravimetric analysis coupled with gas-chromatographic separation and mass-spectrometric characterization (TGA/GC/MS), allowing a precise characterization, establishing clear spectroscopic fingerprints to understand the functionalization protocol in CNOs. This work provides a better insight of potassium intercalation in CNOs and the ulterior functionalization by different organic species.

## Results and Discussion

**Figure 1:**
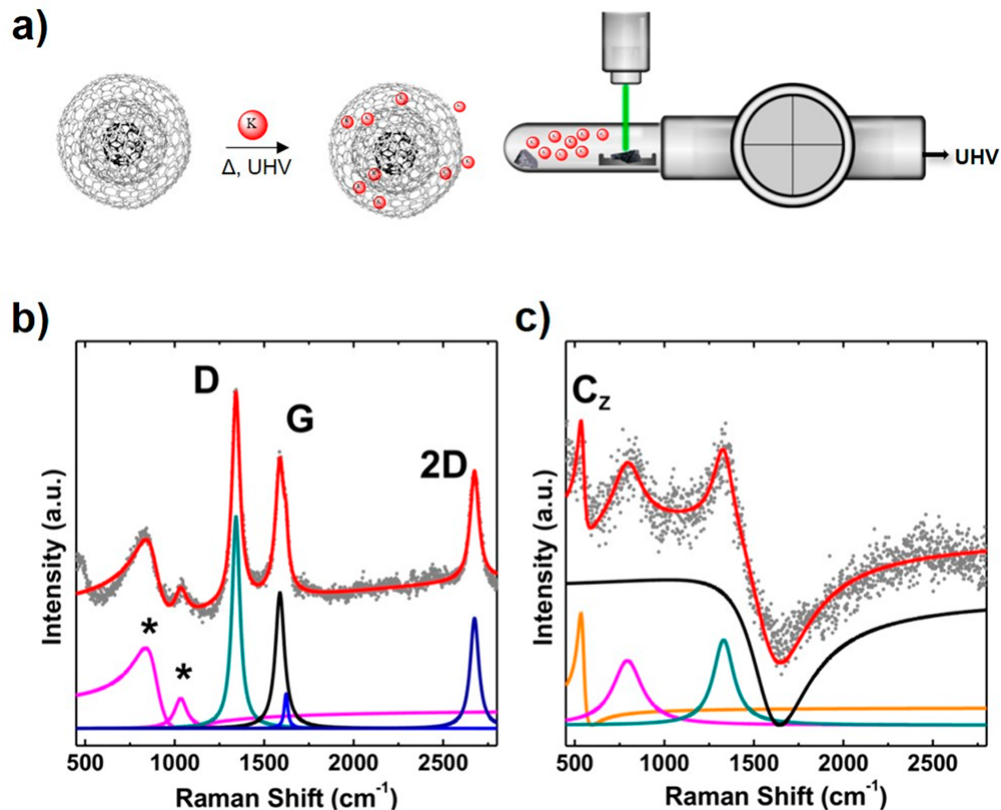
In situ Raman spectra recorded under high-vacuum conditions (∼10^–6^ mbar) on intercalated CNOs and their Raman spectra (λ_ex_ = 532 nm). (a) Scheme of the intercalation reaction and the instrumental setup. (b) Deconvolution of Raman spectra of pristine CNOs that are vacuum sealed. Asterisks (pink lines) indicate characteristic glass tube signals. D (1354 cm^–1^), G (1575 cm^–1^), and 2D (∼2680 cm^–1^) bands characteristic for CNOs depicted in green, black, and blue, respectively. (c) Deconvolution of Raman spectra of K-CNOs. The Raman spectral lines from pristine CNOs evolve along intercalation into: C_Z_ band (orange line ∼500 cm^–1^), glass tube peak (pink line), D (green line), and Fano-like G (black).

The first objective of our work is to investigate the effects of intercalation in CNOs by the intrusion of metallic potassium. The reductive approach via potassium intercalation has already been widely investigated on several carbon nanomaterials, such as graphene, graphite, fullerenes, and carbon nanotubes,^36−43^ as well as other postgraphene materials such as black phosphorus or molybdenum disulfide.^44−47^ Its advantages in terms of an increase in reactivity and in yield of the reaction have been demonstrated by several studies of interest,^48,49^ paving the way for the translation of this approach to CNOs. To the best of our knowledge, only a few studies suggest the possibility of CNOs intercalation, but without unambiguous demonstration.^30,50^ In one case, Molina-Ontoria *et al.* showed the functionalization of CNOs after a liquid Na/K activation.^30^ But only Šiller and co-workers have specifically investigated this intercalation compound with potassium by XPS spectroscopy^50^ showing that potassium-treated CNOs behave as a graphite nanocrystal (closer to graphene) instead of exhibiting a molecular behavior despite their molecular-fullerene-like structure. Moreover, by means of carbon dioxide treatment at 1020 K, the CNO structure is damaged and further opened, derived from partial oxidation that creates pores to foster the intercalation.^51^ Herein, to pursue the understanding of the intercalation mechanism and final properties, CNO powder was intercalated with metallic potassium at 180 °C for 3 days under an Argon atmosphere (K-CNOs) (Figure 1a, see the Supporting Information (SI) for a detailed procedure). The ratio between K and carbon atoms was about 1:8, following a similar procedure reported to be the optimal ratio for KC_8_ GICs.^39^ Similar to the case of GICs, high temperature and mechanical mixing of the powder are aimed to promote the intercalation of K between the shells of a single onion. It is worth mentioning that the intercalation should proceed through lattice defects on the surface of the CNOs (*vide infra*). The analysis of intercalated CNOs needs to be assessed *in situ*, and due to the high reactivity of this species special controlled conditions for the analysis are required. With this intention, K-CNOs were vacuum-sealed in glass vials, allowing the transportation and the direct analysis of the intercalated compound. *In situ* Raman analysis was performed at different times to follow the occurring change (Figure 1b). Raman spectra of pristine CNOs are typically composed of D (1354 cm^–1^), G (1575 cm^–1^), and 2D (∼2680 cm^–1^) bands (Figure 1b, red line) and evolve with intercalation time (Figures 1c and S3). The G band decreases in intensity, while the 2D band disappears after 72 h. This effect is a characteristic feature of highly doped regimes present in the carbon allotrope, which blocks the possibility of having a double resonance process.^52^ In addition, a new peak appeared at ∼500 cm^–1^ (Figure 1c, red line). This new peak, the so-called C_Z_ band highlighted by the orange line in the deconvoluted spectra of K-CNOs (Figure 1c), is characteristic for potassium intercalated multiwalled carbon nanotubes (MWNTs)^36^ and KC_8_ graphite (537 to 565 cm^–1^ respectively).^53^ This peak arises from the out-of-plane vibration of the graphitic carbon atoms and only becomes Raman active under intercalation, when the K atoms impose a superstructure within the carbon lattice.^36^ Conspicuously, the G line exhibits a Fano resonance formation (Figure 1c, black line) associated with electron–phonon couplings between the K atoms and the graphitic structure originating from the in-plane E_2g_^1^ vibration of the carbon atoms.^54,55^ As observed in Figure 1c, the G-line in the CNOs turned from a Lorentzian line into a Fano-like shape. This effect results from introducing a high concentration of K within the lattice structure of the CNOs. The Fano resonance is a characteristic signature from a highly doped potassium intercalated compound as it has been shown from a stage I GIC KC_8_ in MWCNTs KC_8_.^36^ In addition, it is highly important to consider that an asymmetric Fano line shape of the G-line originates from the strong electron phonon-coupling existing between the K atoms and the graphitic structure of the fullerene and other intercalation compounds. The strong electron phonon-coupling in graphitic based intercalation compounds is a signature of a superconducting potential behavior of the material. In this sense, one can estimate the renormalized electron–phonon scattering line width (γ^EPC^) by measuring the G-line frequency of the Fano line (ω_G_). However, in our case, this is not possible, as the already established values for adiabatic and nonadiabatic phonon frequencies in graphite^56^ will not work for fullerenes as the model may have to be restated for this particular nanostructure.

**Figure 2:**
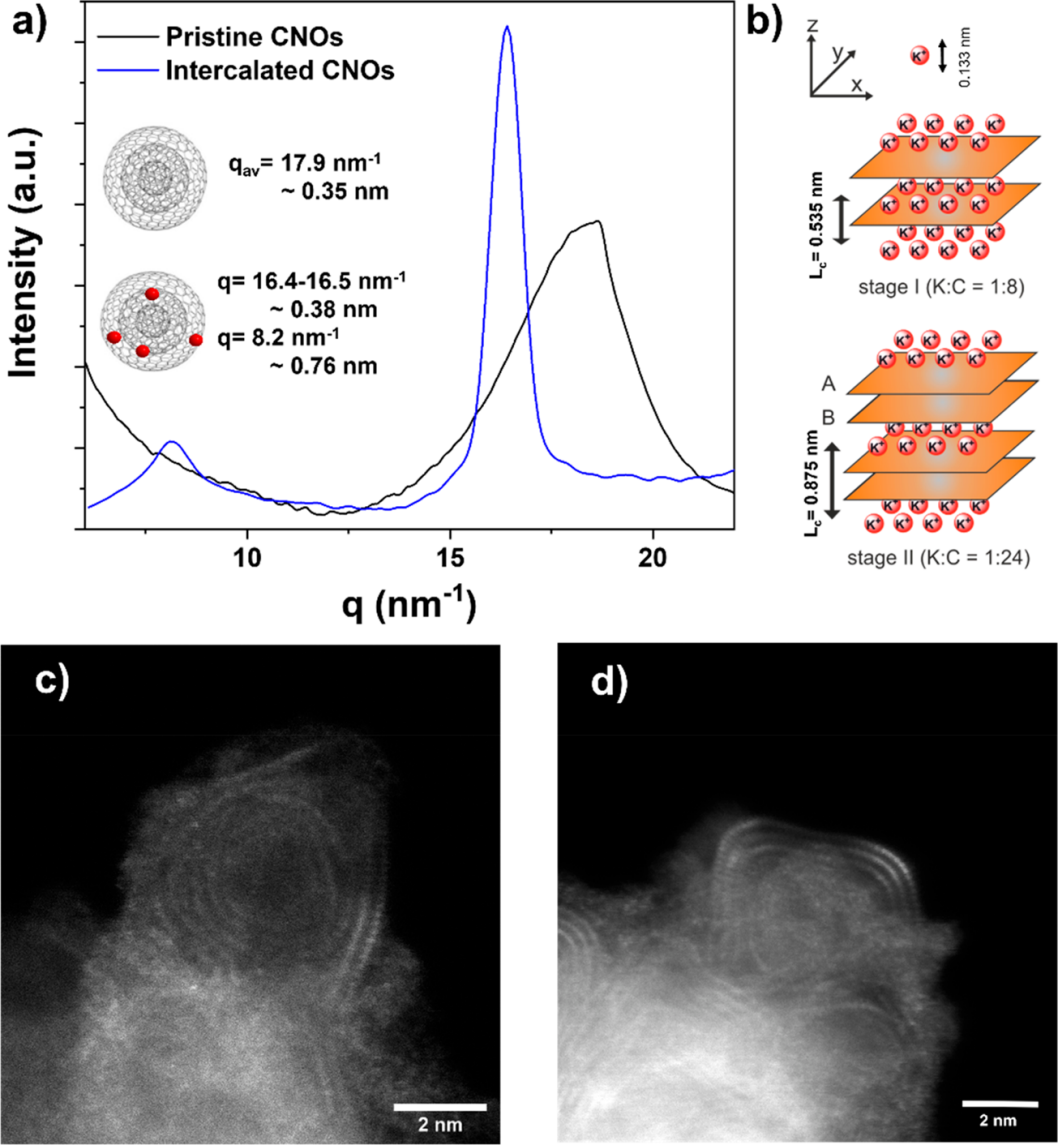
(a) X-ray powder diffraction of pristine and intercalated K-CNOs. Pristine CNOs (black line) are characterized by a broad peak with interlayer distance of ∼0.35 nm (*q*_average_= 17.9 nm^–1^). K-CNOs (blue line) characterized by two major peaks corresponding to interlayer distances of ∼0.38 and ∼0.76 nm (*q* = 8.2 and 16.4 nm^–1^) are shown. (b) Top: scheme of stage I intercalation compound corresponding to K:C = 1:8. Bottom: stage II intercalation compound, corresponding to K:C = 1:24. (c) STEM-MAADF image of pristine CNOs. (d) STEM-MAADF image of intercalated K-CNOs.

This first analysis is confirming that the intercalation treatment is leading to changes in the CNOs’ electronic structure. To further investigate the effects of intercalation in CNOs, we performed a direct observation of the changes in the CNOs structure through XRD and scanning TEM (STEM). All the analyses were performed under vacuum, maintaining the samples under an inert atmosphere to avoid the deintercalation/oxidation of the reduced species (see the SI). XRD analysis of K-CNOs revealed the presence of two dominant interlayer distances, 0.38 and 0.76 nm (Figure 2a). The first value is close to the one of conventional graphite/graphitic carbon but slightly higher than that observed for the pristine CNOs (∼0.35 nm). The second value of *ca.* 0.76 nm reflects the potassium-intercalated onion shells. Comparing this with the family of GICs, the interlayer distance roughly suggests a stage II compound rather than a conventional stage I compound. Specifically, stage I, where the interlayer distance is ∼0.535 nm and corresponding to a ratio between K:C = 1:8 (Figure 2b, top), and stage II, in which ∼0.875 nm corresponds to a ratio of K:C = 1:24, with the coexistence of two interlayer distances: intercalated and nonintercalated ones (Figure 2b, bottom). Additionally, the formation of Daumas–Hérold type defects cannot be ruled out.^57^ The increase of the interlayer distance is a first suggestion of the occurrence of a potassium intercalation, due to the penetration of the metal between the shells. Moreover, the comparison with GICs is suggesting that not all the potassium employed for the intercalation penetrates inside the onions, from which an interlayer distance that lies in between KC_8_ and KC_24_ is obtained.^58−60^ This phenomenon could be explained by the difficulty for potassium to enter the first shell of CNOs, which can only occur via defects, and then diffuse between the other inner shells.

To gain further insights into the structure and morphology of the intercalated CNOs, all samples were characterized using the aberration corrected Nion UltraSTEM 100 dedicated scanning transmission electron microscope in Vienna. The intercalated samples were transported in an inert atmosphere and placed on sample holders inside an argon glovebox. They were then moved in the argon atmosphere to the vacuum system connected to the microscope.^61^ Pristine samples were transferred through air. All images were recorded with an acceleration voltage of 60 kV using the medium angle annular dark field (MAADF) detector. Based on a statistical analysis of STEM-MAADF images (similar to the ones shown in Figure 2c), the average number of shells is around six. Interestingly, instead of being round, the onions typically exhibited ellipsoidal shapes with different degrees of polygonization, which is also observed for CNO obtained by computer models (*vide infra*). These modifications of the structure can locally affect the interlayer distance which plays an important role in the intercalation. There was no apparent difference in the shape of the onions after the intercalation (see Figure 2d for an example), suggesting that, if potassium enters the onions shells, it does not disrupt the layered structure.

**Figure 3:**
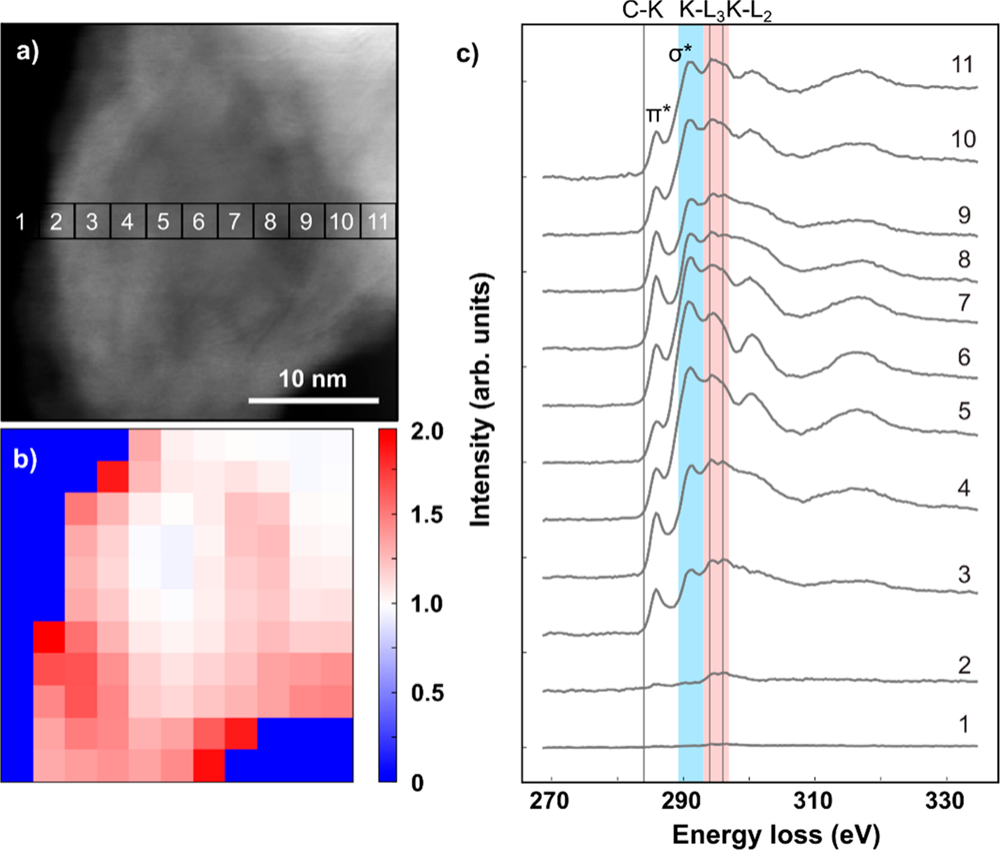
Electron energy loss spectroscopy analysis of an intercalated nano-onion (K-CNO). (a) Spectrum image with each pixel having the intensity corresponding to the sum of the spectrum recorded there leading essentially to thickness contrast. (b) A 11 × 11 pixel map made from the spectrum image of panel (a) by binning the spectra and dividing the integrated intensity within an energy window of 293–297 eV with the intensity from 289–293 eV (except for pixels recorded in vacuum). The energy windows are marked with shaded areas in panel (c). (c) Averaged spectra from the areas marked in panel (a) with numbered squares. Potassium L_3_ and L_2_ peaks are at 294 and 296 eV (inside the pink shaded area), whereas the C-K σ* peak is within the second energy window (area shaded light blue).

Despite *Z*-contrast, the STEM-MAADF images did not allow us to unambiguously locate potassium within the structures. Therefore, we also employed electron energy loss spectroscopy and spectral mapping within the same instrument. A spectral map of a larger nano-onion is shown in Figure 3a (each pixel shows the total intensity of the spectrum recorded at that location, which roughly corresponds to a *Z*-contrast image). The spectra were recorded with a dispersion of *ca*. 0.33 eV/pixel (512 pixels in total) and were centered at 300 eV. Figure 3b shows a map where the original spectrum map was binned from 256 × 256 to 11 × 11 pixels, and for each pixel the signal corresponding to an energy window of 293–297 eV was divided by that from 289–293 eV (pixels with very low signal corresponding to vacuum were set to zero). As can be seen from the spectra in Figure 3c, that correspond to the marked locations in Figure 3a, the first energy window (pink shaded area) contains both potassium peaks K-L_2_ and K-L_3_,whereas the second energy window (light blue shaded area) corresponds to the σ* peak of the carbon K edge. Correspondingly, the red areas in Figure 3b (mostly between the outer layers of the onion) correlate to locations with a high potassium concentration. Notably, in several of the shown spectra, the two potassium peaks are clearly visible (for example, in 3, 4, 8, 9, 10 and 11) despite the overlap with the carbon K peak. Overall, these results support the observation of partial intercalation of CNOs. Additionally, as previously shown by XRD analysis, EELS also suggests an intercalation ratio K:C closer to 1:24, with the remaining potassium being free around the onions.

**Figure 4:**
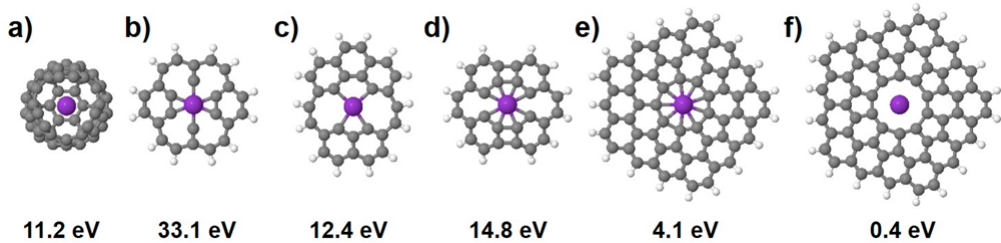
Transition states and corresponding activation energies of K entering C_60_ (a) and trespassing coronene (b), heptacoronene (c), and circulenes with a central octagon (d), nonagon (e), and decagon (f).

Once the intercalation of potassium in CNOs has been demonstrated, we used DFT computer models to explore the different energetics of the intercalation process. For this, we simulated the transition states for insertion of K through polyaromatic hydrocarbons with central rings of increasing sizes ranging from 6 to 10 as well as inside a C_60_ fullerene, Figure 4. The activation energy was computed at the M06-2X-6-31G(d) level with the Gaussian09 software.^62^

The comparison of the mechanism and the energies between the insertion through coronene (33 eV) and fullerene C_60_ (11 eV) through a window opening mechanism is illustrative of how the different curvature and local bonding structures might play a significant role on the activation energy, especially for smaller rings. Nevertheless, these results indicate that only, for relatively large pores, with rings of ≥10 bonds and 0.48 nm in diameter, the insertion barrier for K is low enough (0.4 eV) for the process to proceed at low temperatures.

As previously mentioned, CNOs are often reported as spherical particles, due to their matryoshka-like structure, similar to fullerenes. In contrast, STEM images show different structures such as deformed spheres or ellipsoids. The origin of these modifications remains unclear and has been traditionally attributed to either defects, tension, chemical bond distribution, dimension of the onions, or even temporary deformations as a consequence of the “breathing” of the structures under the electron beam.^63^ To better understand this observation, we carried out a computational study where we, through simulated annealing dynamics, transformed 40 different all-carbon nanodiamond particles ranging from C_35_ to C_969_ to CNOs.^64^ For this, simulations were run with the Brenner bond order potential implemented in a home version of the TINKER code during 2 ns runs with linearly increasing temperatures from 1300 to 3500 K.^65^ Here, 2000 snapshots were taken from each dynamic trajectory and subsequently minimized to find the most thermodynamically stable nanoparticle for each system. In the last step, the so-obtained molecular structures were minimized with the GFN2 extended tight-binding methodology which allows for the efficient computation of large molecular systems with quantum mechanical Hamiltonians.^66^

**Figure 5:**
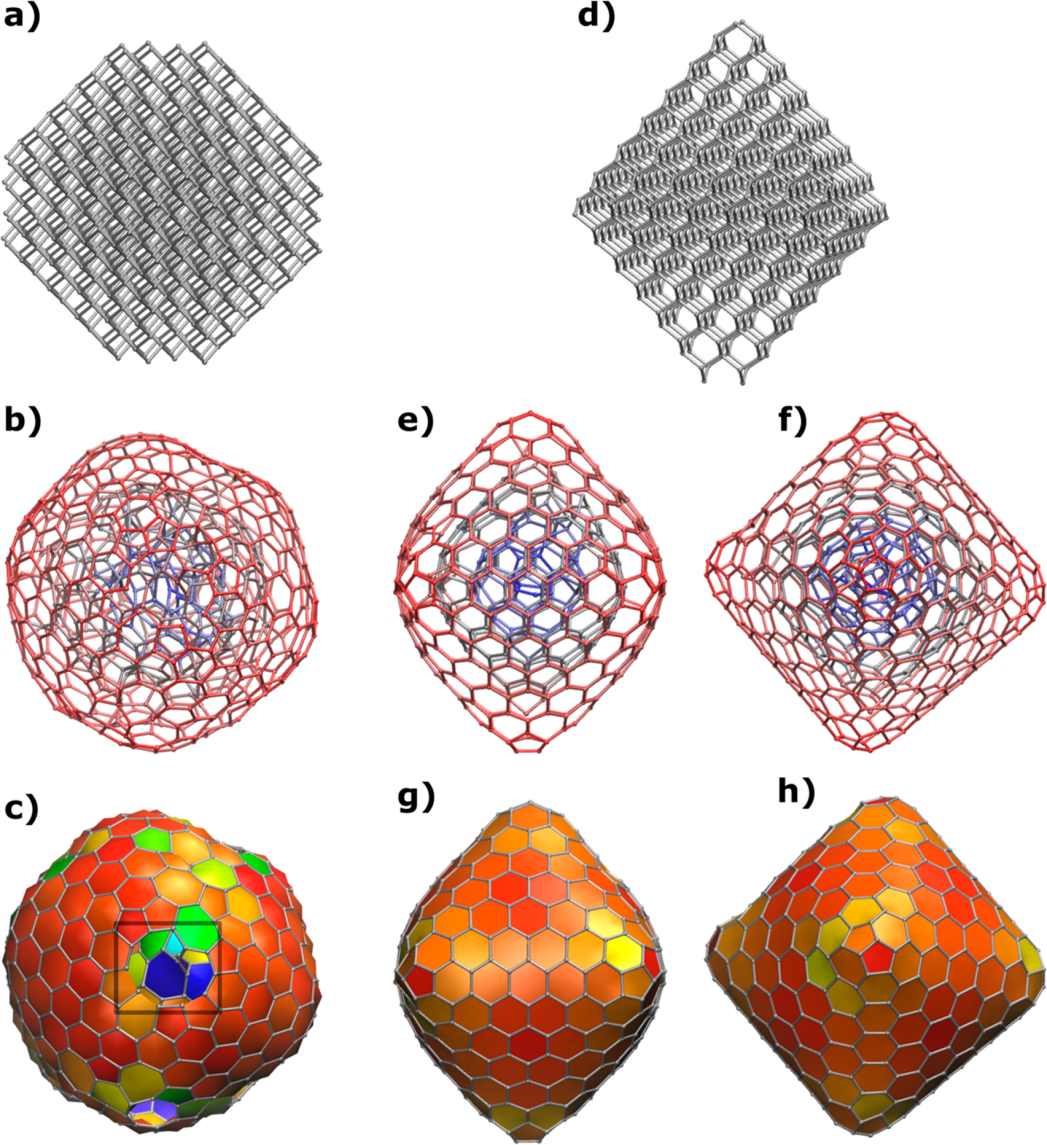
Transformation from nanodiamond to three layer CNOs through simulated annealing. C_753_ nanodiamond (a) and three-layer CNO in two representations (b, c) and C_674_ nanodiamond (d) and three-layer CNO in two representations and two perspectives (e–h). The rectangle in C_753_ CNO (c) serves to highlight the undecagon hole on the CNO surface.

Two representative systems comprising a pseudospherical CNO, C_753_, and a polygonal one, C_674_, were selected and will be discussed comparatively. In both cases, the transformation started by reconstructing the external, and more undercoordinated, region of the initial nanoparticles. The pure octahedral C_674_ nanodiamond kept its regular shape, while the truncated octahedral C_753_ nanodiamond transformed into a pseudospherical CNO (Figure 5). This difference was also reflected by the different simulated annealing temperatures needed: 2093 and 2804 K, respectively. Interestingly, the C_674_ CNO shows a nearly octahedral shape with a defect-free external shell composed exclusively of hexagons and pentagons matching what is found in most fullerenes. In contrast, C_753_ CNO possesses an irregular pseudospherical shape with pentagons, hexagons, and heptagons together with some larger holes with sizes up to undecagons which would be the most likely entry point in this case for the K in the intercalation mechanism. Altogether, these findings confirm the suggestion by Šiller and co-workers that the intercalation of CNOs occurs due to the presence of surface defects, as a result of the synthetic CNO preparation, and strongly support the experimental results shown here.^50,63^

**Scheme 1:**
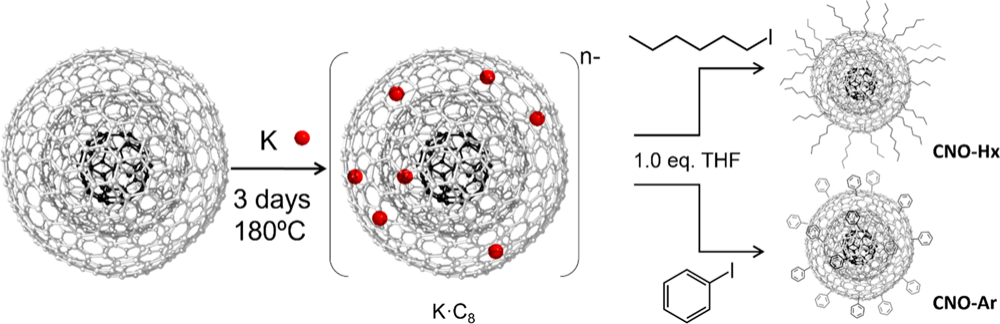
Reaction Scheme of CNO Intercalation (First Step) followed by Functionalization with Hexyl Iodide or Phenyl Iodide (Second Step) and Schematic Representation of Functionalized CNOs

Finally, to prove the efficiency of the potassium intercalation strategy, K-CNOs were functionalized using two reactive species, hexyl iodide and phenyl iodide, well-known and investigated for the functionalization of GICs (Scheme 1, SI for reaction conditions). Both reagents allow a covalent bulk functionalization with the employment of a low amount of reacting species, avoiding the occurrence of side reactions and the formation of coupling products.^39^ Moreover, the choice of well-known reagents allows an easy characterization of the onions due to the simple moieties introduced on their surface.

**Figure 6:**
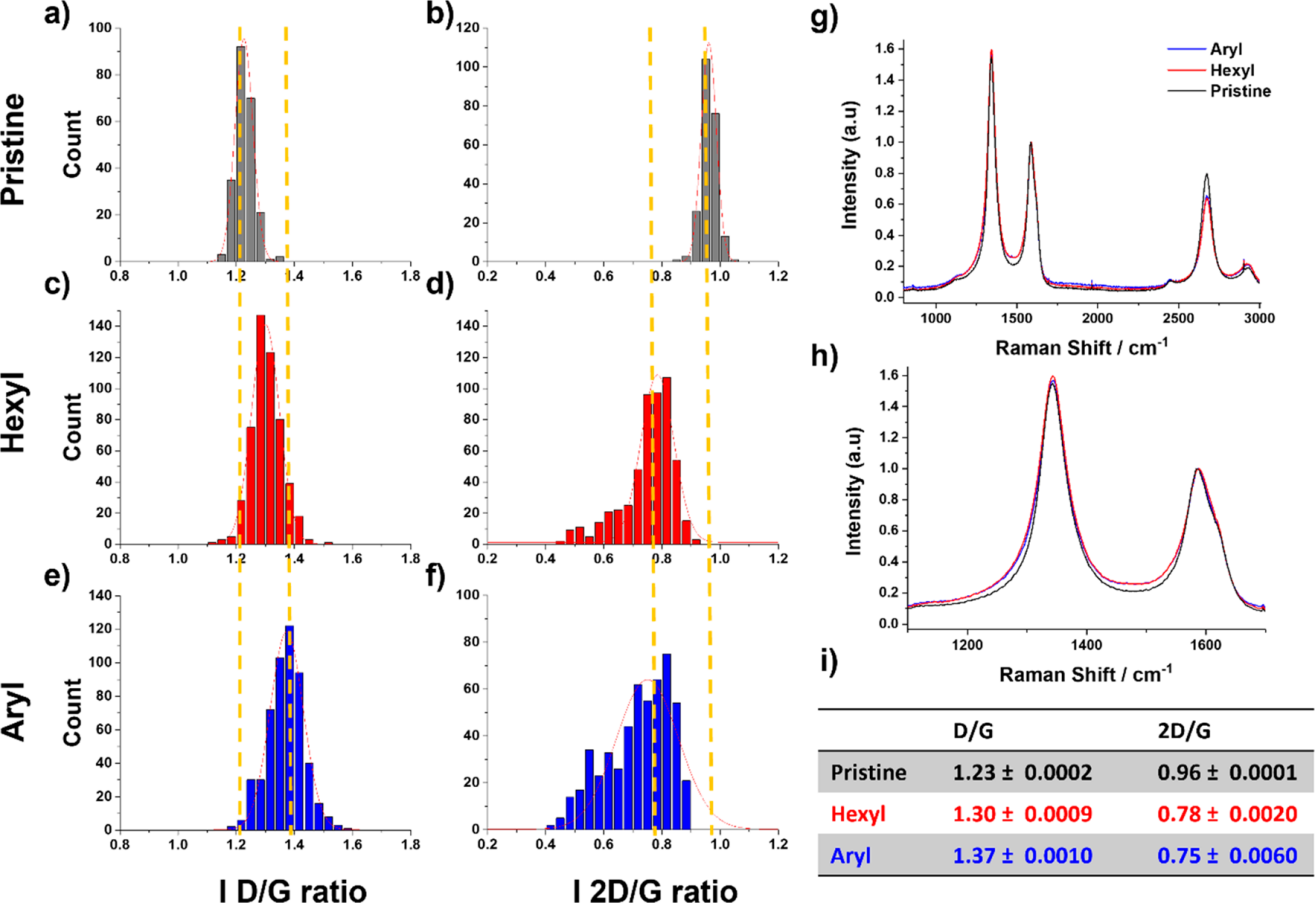
Statistical Raman analysis performed on a 10 μm surface area. First row: Pristine CNOs, 200 points collected for each map., (a) *I*_D_/*I*_G_ ratio and (b) *I*_2D_/*I*_G_ ratio. Second row: Hexyl functionalized CNOs, 625 single points collected, (c) *I*_D_/*I*_G_ ratio and (d) *I*_2D_/*I*_G_ ratio. Third row: Aryl functionalized CNOs, 625 single points collected, (e) I_D_/I_G_ ratio and (f) *I*_2D_/*I*_G_ ratio. (g) Comparison of the mean Raman spectra from the Raman mapping, CNOs pristine (black line), CNOs-hexyl (red line), and CNO-aryl (blue line). (h) Higher magnification of the Raman spectra shown in (g) . (i) Table summarizing the *I*_D_/*I*_G_ ratio and *I*_2D_/*I*_G_ ratio.

Differently from classic organic synthesis, the characterization of nanomaterials requires a cross-correlated analysis of several techniques to prove an intended functionalization. Following this line, we evaluated the number of functional groups and their integrity. Raman spectroscopy is one of the most commonly employed techniques to analyze modifications on the nanomaterials lattice.^67−69^ Similar to graphene, Raman bands such as the D band (∼1350 nm), G band (∼1580 nm), and 2D band (∼2680 nm) are characteristic for CNOs. In contrast to graphene, in which the information about functionalization, number of layers, and surface change can easily be extracted, for CNOs, the presence of an intense D band makes a precise estimation of the *I*_D_/*I*_G_ ratio quite difficult. Moreover, the analysis of hundreds of single point spectra of pristine CNOs reveals a broad distribution of *I*_D_/*I*_G_ ratios ranging from *ca*. 1.1 to 1.4, pointing out the importance of a statistical assessment for the correct interpretation of the functionalization reactions. Indeed, D and G band intensities are very close for onions synthesized by thermal annealing, reducing the change on the spectra due to chemical functionalization. Herein, we performed a unique statistical Raman analysis and temperature dependent Raman analysis on functionalized CNOs (f-CNOs) in order to obtain an accurate estimation of the functionalization reaction. Areas of 10 μm were analyzed, obtaining more than 200 points for the pristine material and more than 600 points for f-CNOs. Figure 6 shows the results of the *I*_D_/*I*_G_ ratio and *I*_2D_/*I*_G_ ratio. The average values of the pristine and the f-CNOs do not show a highly significant difference, with 1.23 ± 0.0002, 1.30 ± 0.0009, and 1.37 ± 0.0010, respectively, for pristine, hexyl, and aryl functionalized onions (see Figure 6, table). However, clear evidence of the functionalization that occurred is shown by the *I*_D_/*I*_G_ and *I*_2D_/*I*_G_ ratio distribution. The first column shows a shift on the *I*_D_/*I*_G_ ratio distribution for both hexyl and aryl functionalized CNOs compared to the pristine onions, with an increase of the intensity of the D band, typically attributed to the functionalization of carbon nanomaterials. Accordingly, the *I*_2D_/*I*_G_ ratio of functionalized CNOs shows a significant shift to lower values and broadening with respect to the pristine one. These observations highlight the importance of the statistical Raman analysis for the characterization of nanomaterials. In fact, from the simple comparison of the average Raman spectra (Figure 6g, h), the information about the intensity ratio would have been lost, resulting in the misleading conclusion that no reaction occurred on the material. Additionally, the analysis of few Raman spectra could have led to errors as well, because of the low number of cases analyzed.

**Figure 7:**
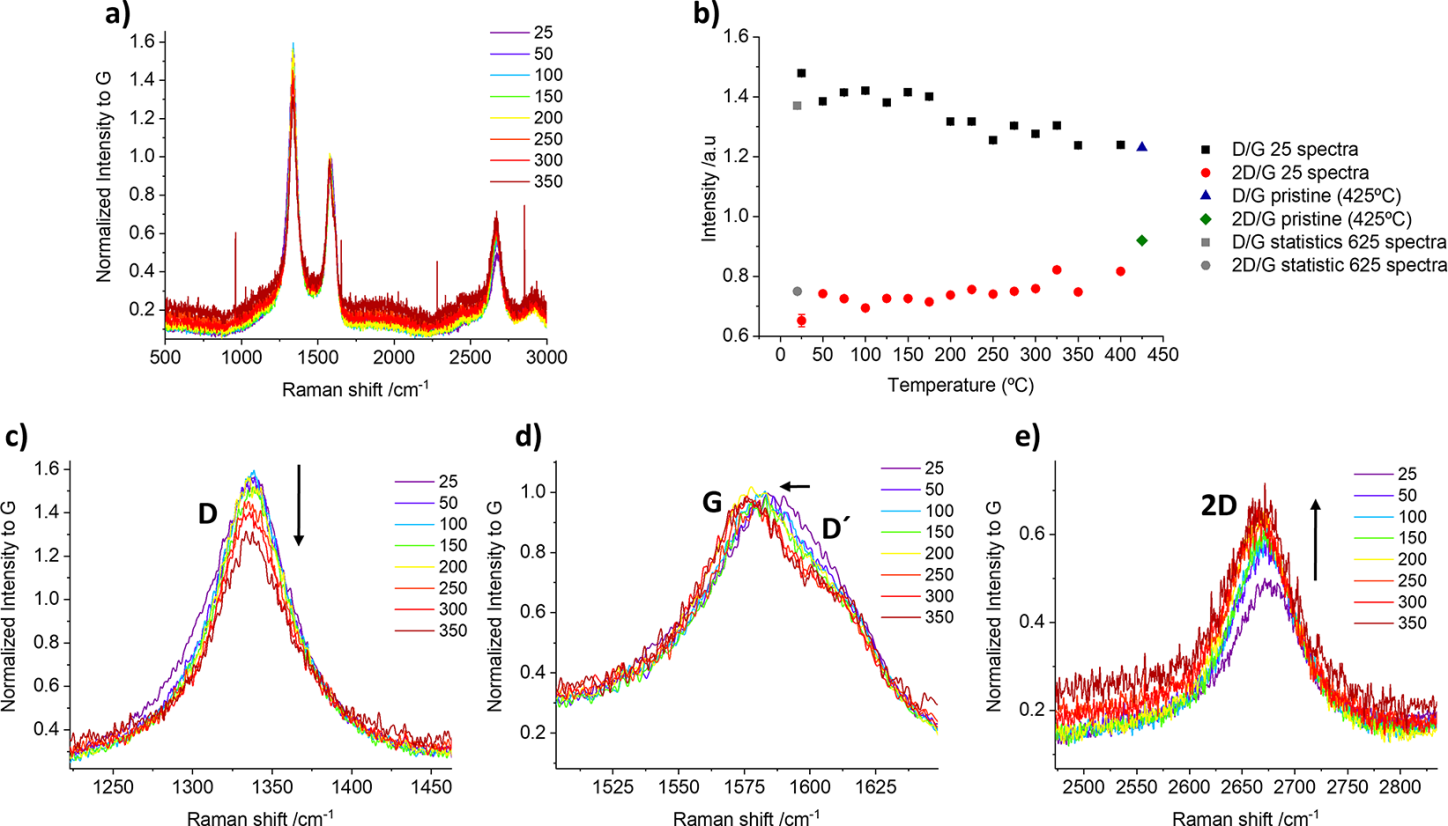
Temperature-dependent Raman analysis of CNOs-aryl. (a) Stacking of Raman spectra from 25 to 350 °C. (b) Evolution of the *I*_D_/*I*_G_ and *I*_2D_/*I*_G_ ratios with increasing temperature. The ratios of the statistical map for pristine CNOs (represented by the blue triangle and the green rhombus) at 425 °C are recovered after the thermal defunctionalization treatment. (c) Magnification of CNOs D band; the decrease of D band intensity with increasing temperatures is highlighted by a black arrow. (d) Magnification of CNOs G and D′ bands, showing D′ disappearance with increasing temperatures. (e) Magnification of CNOs 2D band, showing the increase of 2D band intensity with increasing temperatures.

Furthermore, we performed a temperature-dependent Raman analysis, from 0 to 425 °C, to investigate the reversibility in the functionalization of CNOs. This technique was already employed by our group to understand the response of functional groups and nanomaterials to temperature.^39,48,70^ Starting from 0 °C, a Raman map was collected by 25 °C steps. In this case, the scanning and warming time was minimized, performing maps of 25 single points each, to avoid the prolonged warming of the sample at high temperatures. The results are depicted in Figure 7 for CNOs-aryl (Figures S4 and S5 for pristine and CNOs-hexyl, respectively). A concomitant narrowing and decrease of the D band upon defunctionalization and an increase of intensity of the 2D band, corroborating the defunctionalization due to the increase of the temperature, were observed. Furthermore, the disappearance of the D′ shoulder at around 1620 cm^–1^ is observed upon heating. Finally, and perhaps most importantly, the recovery of the pristine CNOs’ *I*_D_/*I*_G_ ratio after thermal treatment demonstrates that the carbon lattice is not damaged during the reduction or the functionalization process. A reference map analysis on pristine CNOs was also performed to discard possible effects due to temperature. As expected, no significant changes in the Raman spectra were found.

**Figure 8:**
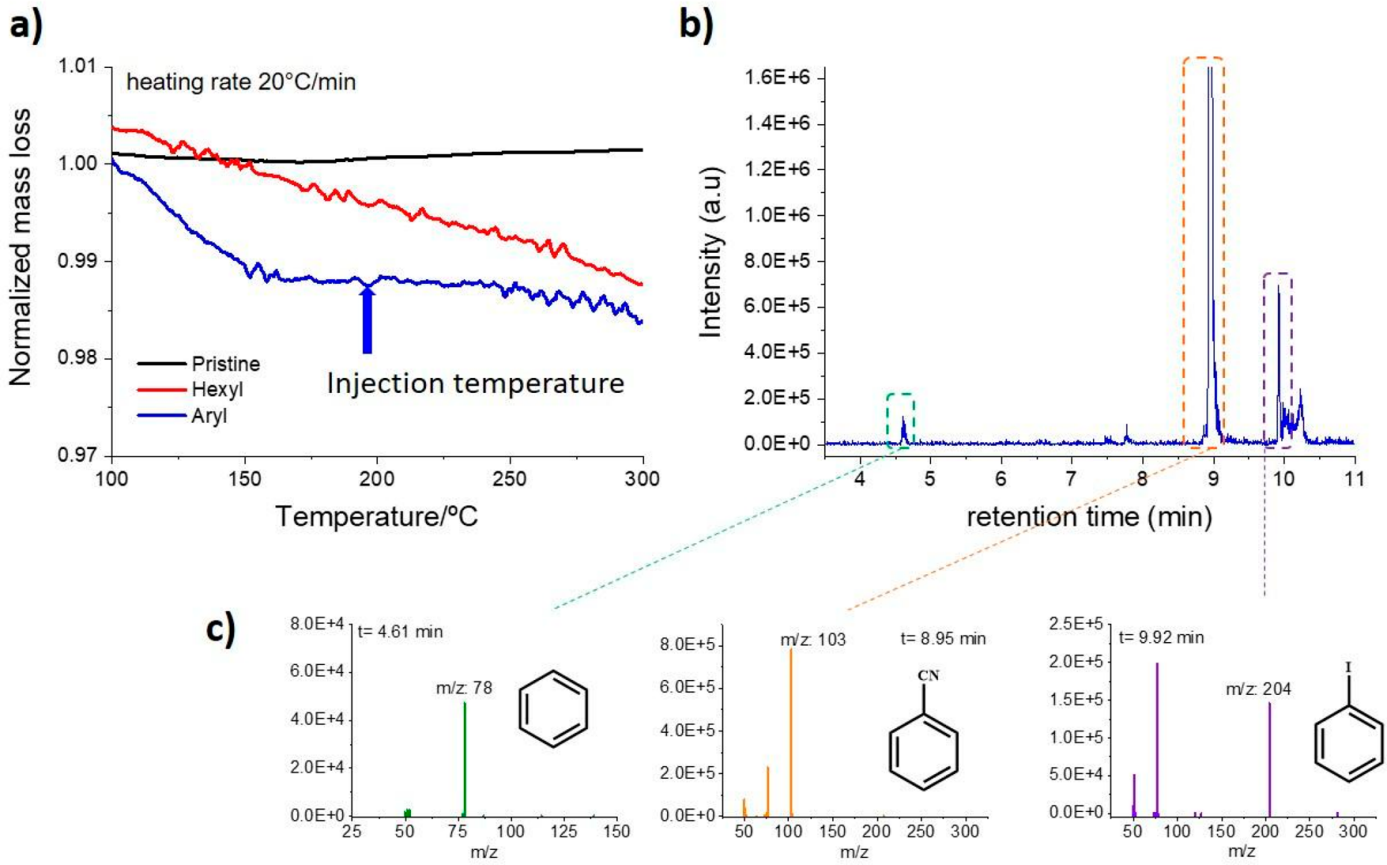
TGA-GC-MS analysis from CNOs-aryl. (a) TGA profile of CNO-aryl (blue line); blue arrow indicates sample injection at 200 °C during TGA analysis. (b) CNOs-aryl GC chromatogram; peaks marked with a colored box are relative to fragments from functional groups. (c) MS fragment analysis of GC peaks at 4.61, 8.95, and 9.92 min. Residual benzonitrile presence from the workup protocol is shown by the peak at ∼8.95 min.

To analyze the fragments of the organic molecules detached from the samples upon heating, a special technique based on the coupling of thermogravimetric analysis, gas chromatography, and mass-spectrometry (TGA/GC/MS) was performed. This technique is known for its efficacy in the characterization of other carbon nanomaterials, even in the presence of multiple functional groups.^70^ Several advantages arise from the combination of these techniques: first, the possibility of choosing the temperature at which to analyze the gas developed and the precise separation of the components through gas chromatography. Second, the high sensitivity of mass spectrometry and cross-reference with mass-fragmentation libraries allow a deep understanding of the composition of each eluted compound, in direct correlation to the defunctionalization of the materials. Initially, simple TGA analysis was carried out (Figure 8a and entire TGA profile in Figure S6a), showing the high stability of pristine CNOs up to a temperature of 700 °C. The CNO-hexyl presents a constant weight loss starting from a temperature of ∼120 °C, while CNO-aryl presents a more marked weight loss at ∼140 °C and then from 200 °C (Figure S6b). Therefore, we collected and analyzed the gas produced at 200 °C for CNO-aryl (Figure 8b) and 180 °C for CNOs-hexyl (Figure S7b). The CNO-aryl sample presents the main peak of benzene after 4.61 min, corresponding to *m*/*z* of 78 (Figure 8c). Together, at 9.92 min, a peak corresponding to unreacted iodobenzene is present (*m*/*z* = 204). A similar behavior was observed by the CNO-Hexyl sample, with three main peaks at 4.14, 4.18, and 5.99 min (Figure S7c). The fragmentation analysis corresponds to different fragments arising from the hexyl chain during the warming of the materials. A similar behavior was shown by conventional TGA-MS direct analysis performed on CNO-hexyl (Figure S8b). The temperature/ion current spectra show three main fragments formed at ∼300 °C during heating of the CNOs. The *m*/*z* fragments of 43, 57, and 86 correspond to linear aliphatic compounds from C_3_H_7_, C_4_H_9_, and C_3_H_7_–CO–CH_2_^+^. The high temperature at which the fragments are detected (>300 °C) confirms their origin as a functional group of CNOs, discarding adsorbed organic molecules, expected to appear at lower temperatures. Other common characterization techniques such as attenuated total reflectance Fourier-transformed infrared spectroscopy (ATR-FTIR), absorption spectroscopy (UV–vis), and dynamic light scattering (DLS) corroborate our results and can be found in the Supporting Information (see SI Figures S9 and S10).

## Conclusion

In this study, we carried out a combination of different *in situ* and *ex situ* characterization techniques supported by extensive theoretical calculations. This demonstrates that CNOs can be successfully intercalated with potassium through surface defects by means of thermal heating at relatively low temperatures. Moreover, we developed the reductive covalent functionalization of CNOs with both hexyl iodide and aryl iodide, showing unambiguous spectroscopic fingerprints thanks to the cross-analysis of statistical Raman spectroscopy and TG-GC-MS, among others. This work provides an important insight into the structure of intercalated and functionalized CNOs as well as the understanding of the basic principles of their alkali metal intercalation and reductive functionalization. This work may lay the foundations for future applications of CNOs in which metal intercalation and surface engineering play a crucial role.

## Supporting Information Available

Synthesis of materials, functionalization, characterization techniques, and Raman statistical analysis (PDF)
