# Supplementary material for: Carbon Nano-onions: Potassium Intercalation and Reductive Covalent Functionalization
Source: J Am Chem Soc. 2021 Oct 26;143(45):18997–9007. doi: 10.1021/jacs.1c07604 (PMC8603384; doi:10.1021/jacs.1c07604)
Supplement: Supplementary file 1 — ja1c07604_si_001.pdf [file ja1c07604_si_001.pdf]

Supporting information

Carbon Nano-onions: Potassium Intercalation and Reductive Covalent Functionalization

M. Eugenia Pérez-Ojeda,[a,b,*] Edison Castro,[c] Claudia Kröckel,[a,b] Matteo Andrea Lucherelli,[d] Ursula Ludacka,[e] Jani Kotakoski,[e] Katharina Werbach,[e] Herwig Peterlik,[e] Manuel Melle-Franco,[f] Julio C. Chacón-Torres,[g] Frank Hauke,[a,b] Luis Echegoyen,[c,*] Andreas Hirsch[a,b,*] Gonzalo Abellán,[d,*]

[a] Department of Chemistry and Pharmacy, Friedrich-Alexander University of Erlangen-Nuremberg, Chair of Organic Chamistry II, Nikolaus-Fiebiger-Str. 10, 91058 Erlangen, Germany. andreas.hirsch@fau.de, eugenia.perez-ojeda@fau.de

[b] Joint Institute of Advanced Materials and Processes (ZMP), Friedrich-Alexander University of Erlangen-Nuremberg, Dr.-Mack-Str. 81, D-90762 Fürth, Germany.

[c] Department of Chemistry, University of Texas at El Paso, El Paso, Texas 79968, United States. echegoyen@utep.edu

[d] Instituto de Ciencia Molecular, Universidad de Valencia, Catedrático José Beltrán 2, 46980 Paterna, Spain. gonzalo.abellan@uv.es .

[e] Faculty of Physics, University of Vienna, Boltzmanngasse 5, 1090 Vienna, Austria.

[f] CICECO-Aveiro Institute of Materials, Department of Chemistry, University of Aveiro, 3810-193, Aveiro, Portugal.

[g] Yachay Tech University, School of Physical Sciences and Nanotechnology, 100119-Urcuquí, Ecuador.

Carbon nano-onions, Fullerenes, covalent functionalization, Raman spectroscopy, intercalation compounds, DFT

Raman analysis 2
Statistical Raman Spectroscopy 2
UV-vis 3
FTIR 3
STEM and EELS 3
In Situ RAMAN 3
X-ray diffraction (XRD) 4
Dynamic Light Scattering. 4
Thermogravimetric characterization 4
Experimental Section 5
Carbon nano-onions synthesis 5
CNO-intercalation 7
CNO-functionalization 8
Statistical analysis of temperature dependent Raman 16
1.-CNO-Aryl 16
2.-CNO-Hexyl 24
3.- CNO Pristine 32
References 37

Materials and methods

Potassium, hexyl iodide, and benzyl iodide were bought from Sigma Aldrich and used directly in the glovebox without further purification. Benzonitrile anhydrous (PhCN) and THF from Sigma Aldrich were dried over molecular sieves (3 Å) until $H_2O \leq 0.1$ ppm and afterward pump-freeze 5 cycles to completely degas it up to O_2 content ≤ 0.1 ppm. THF was further distilled inside the glovebox. All work-up solvents (H_2O, c-hexane, *i*-propanol, and acetone) used for washing the functionalized samples were previously distilled.

Raman analysis

Raman spectra were analyzed with Labspec software. In all cases only a baseline correction (type: line, grad: 2) was applied. All spectra were normalized to the G band for comparison. Origin software was used for statistical analysis after converting the matrix to xyz-worksheet and applying frequency counts with 60 Bins (bin beginning = 0, bin end = 2). The obtained histograms were fitted to Gaussian functions.

Statistical Raman Spectroscopy

Raman spectra were acquired on a Horiba LabRAM Aramis confocal Raman microscope equipped with an automated XYZ table (Märzhäuser) and a laser spot size of ca. 1 µm (50X objective, NA 0.80). All measurements were conducted using an excitation wavelength of 532 nm, D2 filter (incident laser energy ~0.17 mW), acquisition times of 2s, 3 accumulations and a grating of 1800 grooves mm^{-1}. The spectrometer was calibrated in frequency with diamond (1332 cm^{-1}). Statistical maps with step size of 10 µm were

recorded giving 625 single-point spectra for functionalized samples, 225 spectra for pristine CNO and 25 spectra for the temperature dependent experiments.

Temperature dependent statistical Raman Spectroscopy (t-SRS) were carried out using a Linkam stage THMS 600 equipped with a liquid nitrogen pump TMS94 for stabilizing temperatures under a constant nitrogen flow. The measures were carried out on glass cover slides with a heating rate of 10 °C min^{-1} from 20 to 400 °C trough a 25 °C interval lapse.

Differences in the D/G or 2D/G ratios obtained from the statistical calculations and the ones directly readable from the normalized mean spectra are observed. This is due, on one hand, to the algorithm that Horiba Labspec 6 uses to calculate the mean spectra which is not just an average of spectra but weights more the spectra with higher quality. Meanwhile, for the statistical calculation, all spectra are equally considered. The statistical analysis is therefore a more real analysis of the sample and becomes especially decisive in heterogeneous samples with only slight variations upon functionalization, as it is the case for CNOs. On the other hand, the statistical calculations it is based on the selected regions for each band. The selected bandwidths in this study were 1280 to 1380 cm^{-1} for D band, 1540 to 1630 cm^{-1} for G band and 2630 to 2720 cm^{-1} for 2D band. Furthermore, the baseline correction also causes a slight difference respect to the calculated mean spectra. Anyhow, it is important to mention that these values are relative intensities and that it is not the absolute value what matters but the relative comparison from one to another under the same exact evaluation conditions.

UV-vis

The UV-vis spectroscopic analysis was performed with a Perkin Elmer Lambda 1050 spectrometer at room temperature in sealed quartz cuvettes (Hellma) with 1 cm path length.

FTIR

FTIR spectra were recorded with a BrukerTensor 27 on a ZnSe plate spectrometer. Vibrations are shown in wavenumbers $\tilde{\nu}$ (cm^{-1}).

STEM and EELS

Scanning transmission electron microscopy (STEM) and electron energy loss spectroscopy (EELS) analyses were carried out with the aberration corrected dedicated STEM instrument Nion UltraSTEM 100 in Vienna. This instrument has been modified[1] to allow direct sample transfer between the microscope column and an attached vacuum system, which allows bringing samples to the microscope directly from a protective atmosphere from an argon glove box without exposing them to air, which is especially important for oxygen sensitive samples. The highest pressure in the system is in the range of 10^{-8} mbar, typical pressure inside the sample area of the microscope is $1x10^{-9}$ mbar. The microscope was operated at 60 kV, and the images were recorded with the medium angle annular dark field (MAADF) detector with an annular range of 80-300 mrad. The convergence semiangle of the electron probe is 30 mrad. The EELS acquisition setup consists of a Gatan PEELS 666 spectrometer retrofitted with an Andor iXon 897 electron-multiplying charge-coupled device (EMCCD) camera. The energy dispersion was ca. 0.33 eV/px with a total of 512 px. The individual spectra were background subtracted by fitting power law to the spectrum before the carbon K edge (with an onset at 284 eV).

In Situ RAMAN

In situ Raman measurements were acquired on a Horiba LabRAM Evolution confocal Raman microscope at an excitation wavelength of 532 nm and a laser spot size of ca. 1.3 µm (50x objective, NA 0.50) with an incident laser energy of about 0.8 mW, 180 acquisition time and 3 accumulations, using a grating of 600 grooves mm^{-1}. The spectrometer was calibrated in frequency with silicon oxide (520 cm^{-1}).

X-ray diffraction (XRD)

For XRD, pristine CNOs were placed between two tapes and measured in vacuum in a Bruker Nanostar with CuKα radiation from a microfocus source (Incoatec high brilliance), equipped with a 2D detector (VÅNTEC 2000) for the small-angle X-ray scattering and an image plate (Fuji FLA 7000) for the wide-angle X-ray scattering range. Intercalated CNOs were measured in glass capillaries in a Bruker Smart Apex diffractometer with MoKα radiation to overcome the absorption of the capillaries. All 2D images were radially averaged, corrected for the respective background, and are shown in dependence on the scattering vector $q=(4\pi/\lambda) \sin \theta$, where λ is the respective X-ray wavelength and 2θ the scattering angle.

Dynamic Light Scattering.

DLS measurements were carried out in a Zetasizer Nano series ZEN3600 (Malvern Instruments) provided with a red laser (633 nm). 173° angle (NIBS, "noninvasive back scatter") was used. Three consecutive measures were performed after an equilibration time of 30 s. NMP and THF previously filtered through PTFE 0.2 µm filters were employed. The samples were evaluated at 2.5 mg/mL concentrations as for the UV-vis measurements.

Thermogravimetric characterization

TGA analysis was carried out on a Netzsch STA 409 CD coupled with a Skimmer QMS 422 mass spectrometer (MS/EI). The measurements were implemented from 20 – 700 °C with a 10 K/min gradient. Sample weights of around 3 mg were analyzed under He atmosphere with a gas flow of 80 mL/min. Thermogravimetric analysis coupled to gas chromatographic separation (GC) and a mass spectrometer was also carried out on a Perkin Elmer Pyris 1 TGA instrument using time-dependent temperatures profiles in the range of 20 and 900 °C (20 °K/min gradient) and N_2 gas flow (70 mL/min). The sample weights were around 1 to 2 mg. The evolved gases detached from the sample at 200 °C were then transferred (150 µL) to the GC system through a TL9000 interface at a constant temperature and separated by a GC-Clarus 680 using a polysiloxane-coated Elite-5MS capillary column: 30 cm length, 0.25 mm diameter, 0.25 µm film thickness with the following parameters: injector zone 280 °C, detection zone 250°C, split 8.2 mL/min, N_2 flow rate 10 mL/min, temperature profile = 34 min total run time in dynamic ramp = 24 min 40–280 °C with a 10 K/min gradient followed by an isothermal step of 10 min. MS analysis was performed on MS Clarus SQ8C (multiplier 1800 V) and processed with TurboMass Software and NIST MS search 2.0.

Experimental Section

Carbon nano-onions synthesis

The CNOs were obtained by a heating process at 1650 °C under a helium flow for 2h as shown in Figure S1 and Table S1. Finally, the CNOs were annealed at 400 °C in air for 4 h. The CNOs were characterized by TGA (Figure S2) and Raman spectroscopy (Figure S3).[2]

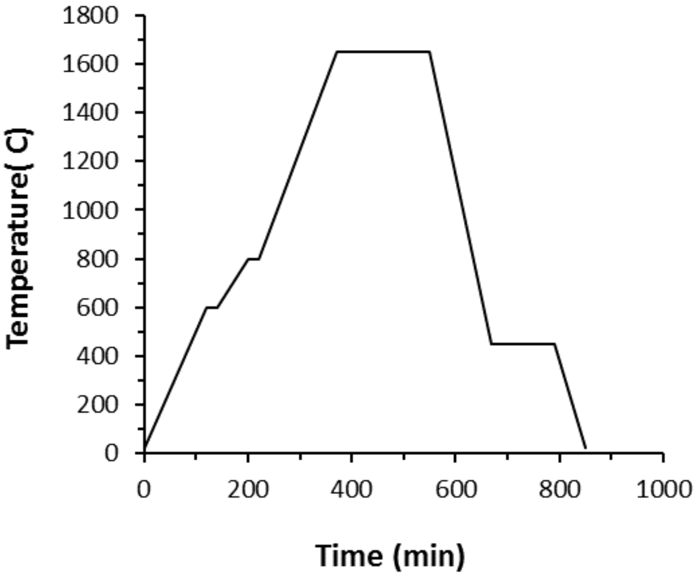

Figure S1. Heating process for the preparation of CNOs.

Time (min)	Temperature (ºC)
0	25
120	600
140	600
200	800
220	800
370	1650
550	1650
670	450
790	450
850	25

Table S1. Ramping conditions

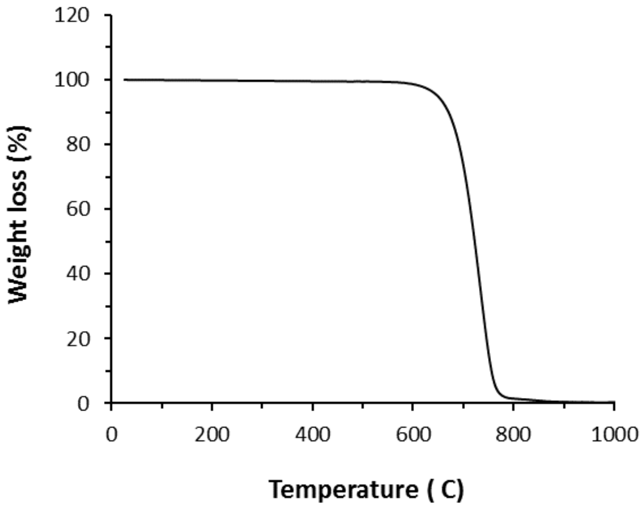

Figure S2. TGA under a N_2 atmosphere and 10 ºC/min of heating rate.

CNO-intercalation

Pristine CNOs were pre-dried under vacuum in an oven at 75 °C overnight and immediately transferred to an argon-filled Labmaster SP (MBraun) glovebox equipped with a gas filter and oxygen and humidity levels ≤ 0.1 ppm. The samples were then heated inside the glovebox in an open vial at 270 °C for 1h. Afterwards, they cooled down and KC_8 stage I intercalated compound was prepared by a solid reaction of CNO (24 mg) and potassium (10 mg) in a glass vial at 180°C for 3 days. No color change was observed due to intercalation.

The sample for the *in situ* doping was annealed under high-vacuum conditions (~ 10^{-6} mbar) at 250 °C overnight and sealed afterwards together with potassium in a glass ampule. The doping was carried out by applying the two-zone vapour process with heating the potassium at about 115 °C while the process was followed *in situ* by recording single-point Raman measurements every minute.

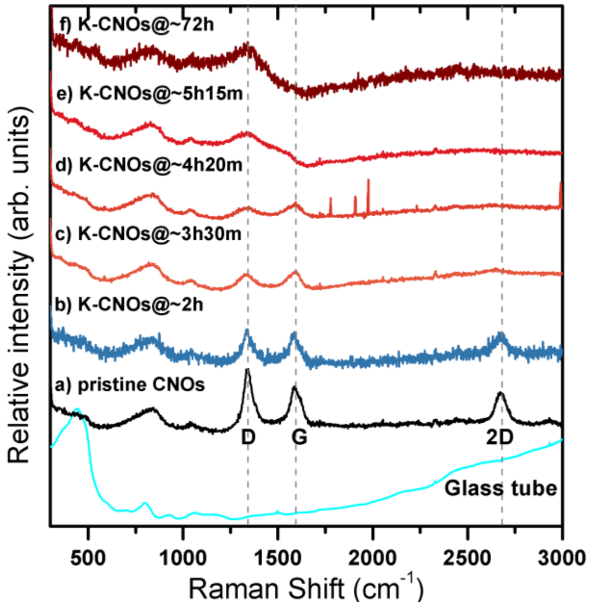

Figure S3: *In situ* Raman doping experiment under high-vacuum conditions. Spectra were collected at λ_{ex}= 532 nm at different intercalation times.

CNO-functionalization

K-CNOs were produced following the general procedure reported above. The powder (34 mg) was cooled down to room temperature, divided into two parts, and dispersed in anhydrous THF (20 mL) using an ultrasonication tip (5 minutes, 40%, 1s on/off). Subsequently, 1 equivalent of the corresponding iodo-derivative was added, and the mixture was stirred inside the glovebox for 24 h at 900 rpm. After this time, benzonitrile was added to check that no residual K^+ ions were present on the crude. No color change was observed due to $PhCN^{\cdot -}$ radical anions, thus confirming that the samples were fully discharged. The workup protocol consisted of quenching with double-distilled H_2O (10 mL) under argon atmosphere, followed by the addition of c-hexane (20 mL) and H_2O (another 10 mL) already exposed to air. After extraction, the samples were washed twice with water (2 x 20 mL) followed by *i*-PrOH. After filtration, the samples were further washed with *i*-PrOH, H_2O, and acetone and finally dried under vacuum. All experiments were carried out in duplicate.

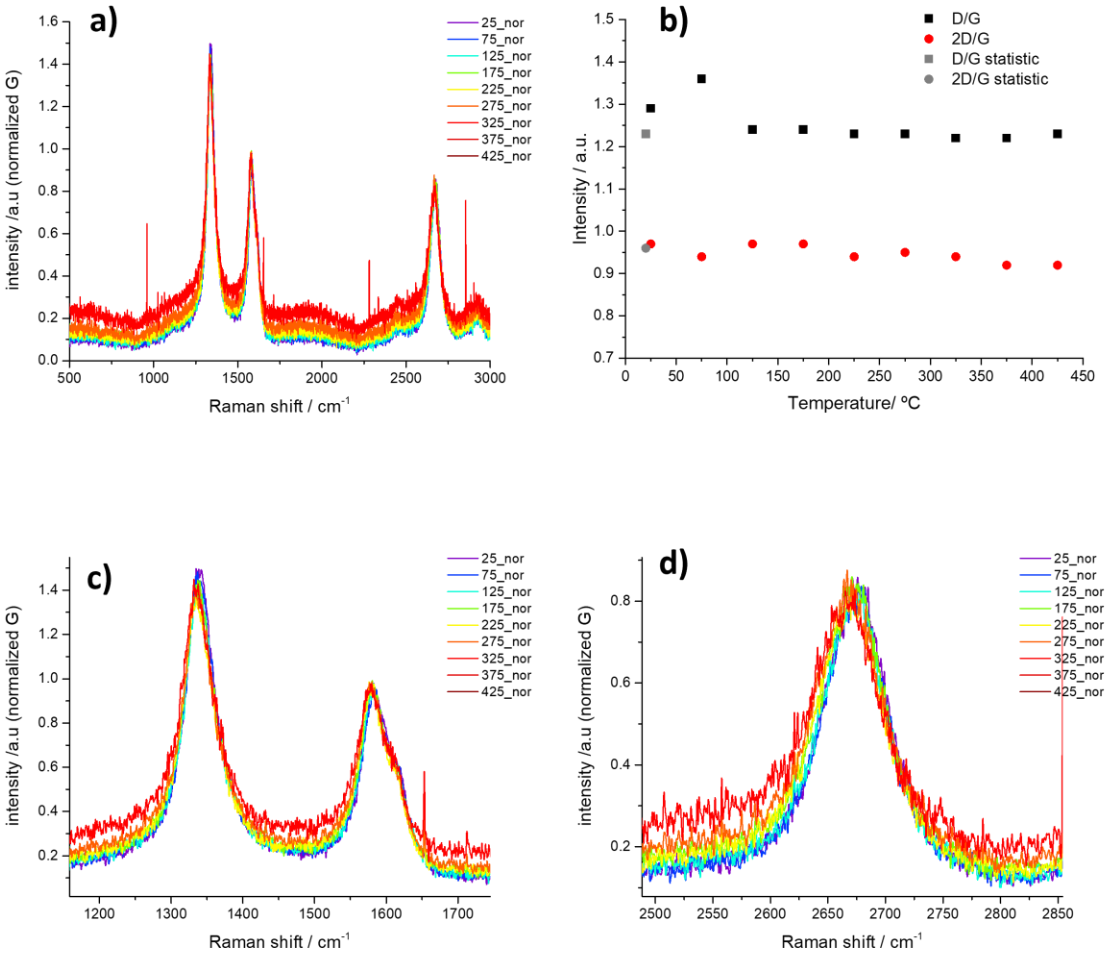

Figure S4: Temperature-dependent Raman analysis of pristine CNOs, from 0 to 425 °C as reference experiment. a) Superposition of all Raman spectra; b) Evolution of the I_D/I_G and I_{2D}/I_G ratios while increasing temperature. c) Magnification of D and G band area; d) Magnification of the 2D band area. No significant changes were observed upon heating.

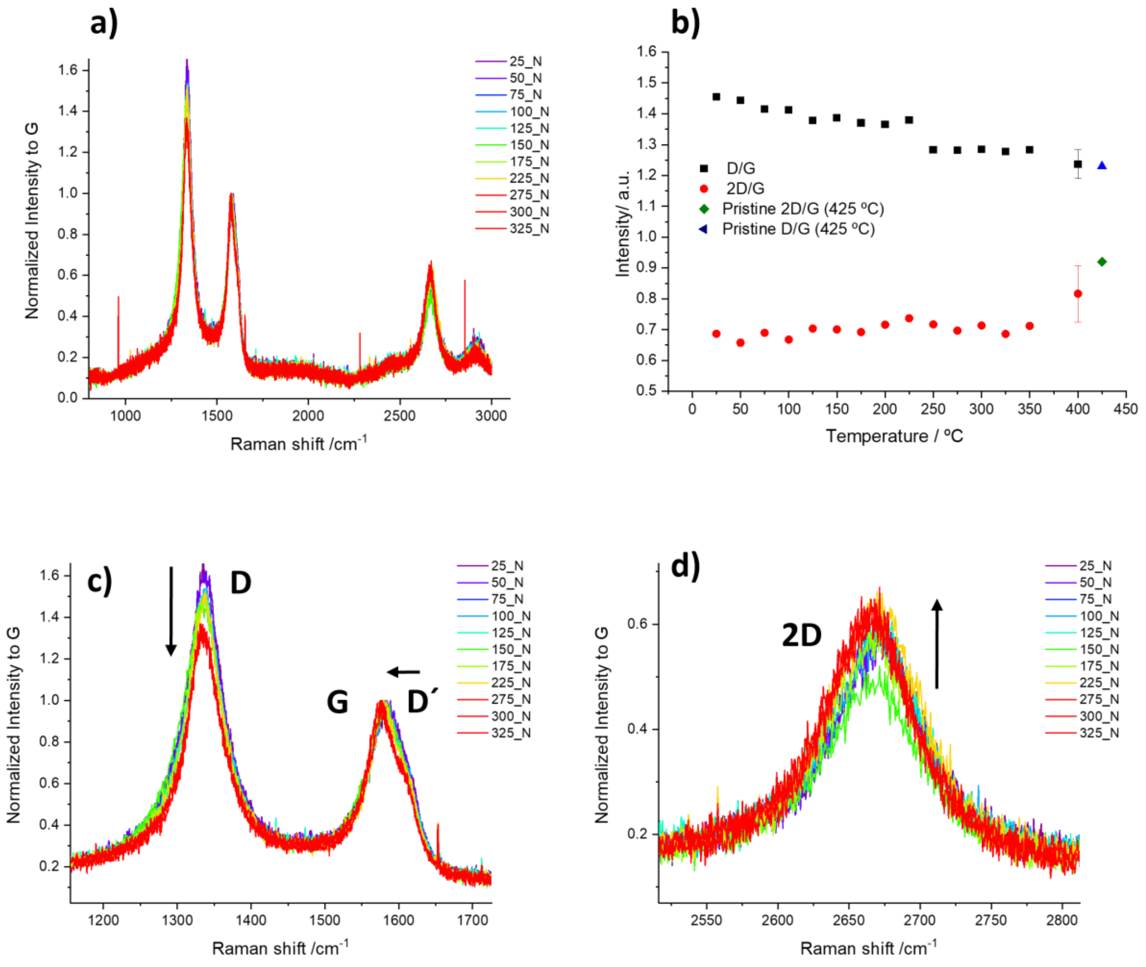

Figure S5: Temperature-dependent Raman analysis of CNOs-Hexyl, from 0 to 325 °C. a) Superposition of all Raman spectra; b) Evolution of the I_D/I_G and I_{2D}/I_G ratios while increasing temperature the ratios of the statistical pristine map are recovered after the thermal defunctionalization treatment c) Magnification of D, D´and G band area; d) Magnification of the 2D band area. The black arrows indicate the main changes observed upon heating.

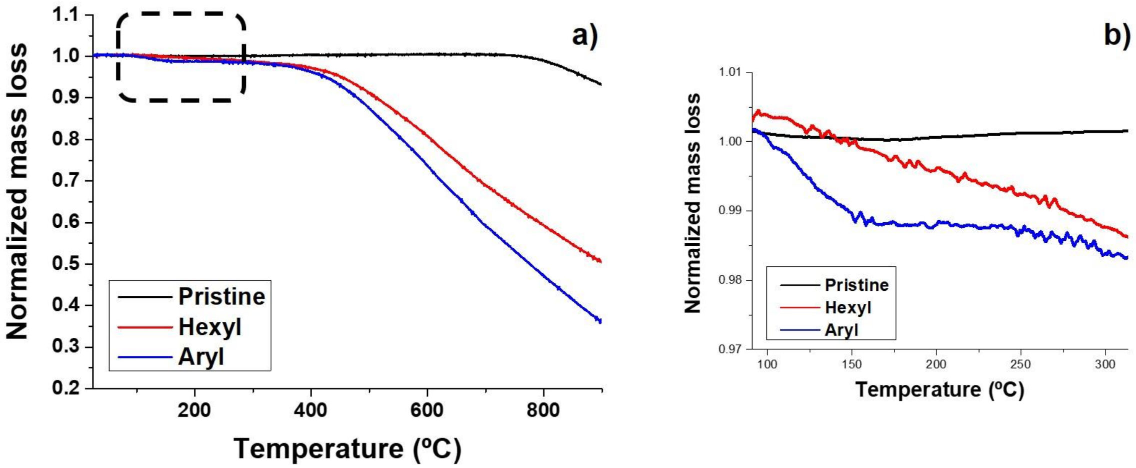

Figure S6: Thermogravimetric analysis of CNOs and functionalized CNOs: a) TGA profile of pristine CNOs (black line), CNO-hexyl (red line), and CNO-Aryl (blue line); b) magnification of the area between 100 and 300 °C. The TGA profile shows a marked weight loss of functionalized CNOs at a temperature of 140-300 °C.

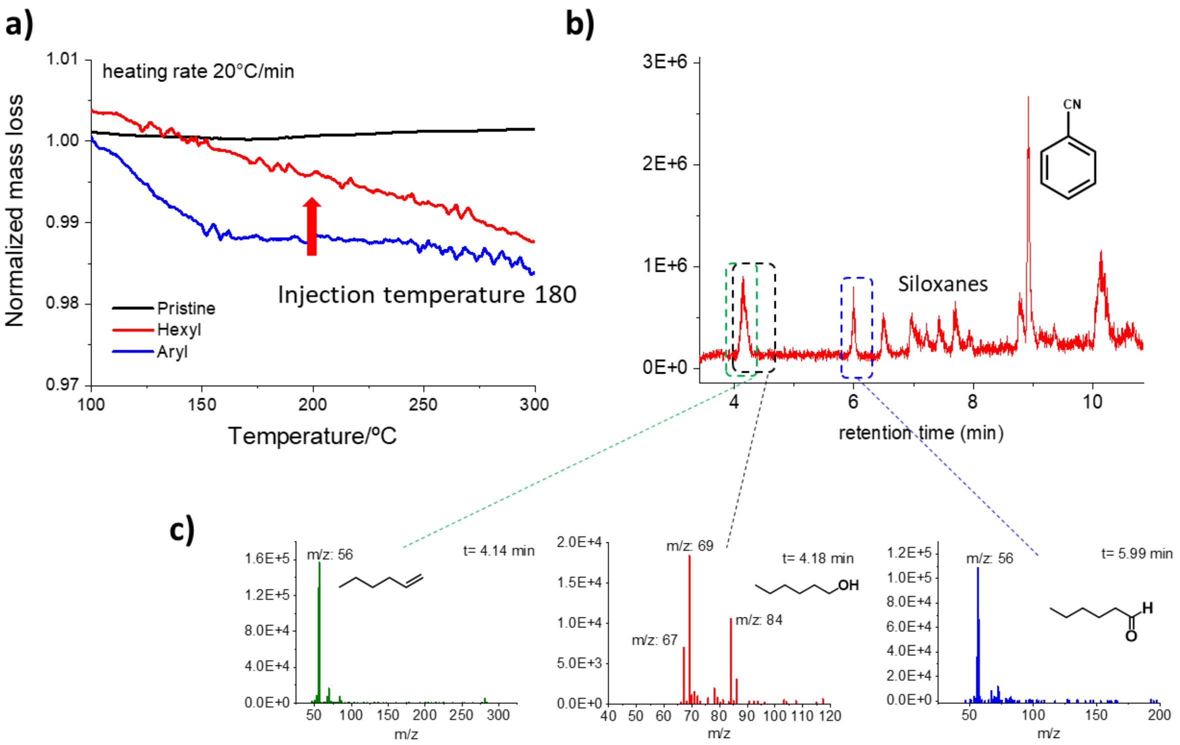

Figure S7: TGA-GC-MS analysis of CNOs-Hexyl a) TGA indicating the injection temperature b) Gas-chromatogram after the injection at 180°C of CNO-Hexyl sample. c) Mass analysis spectra of the main peaks of the chromatogram. The color of the boxes on the chromatogram corresponds to the color of the m/z distributions.

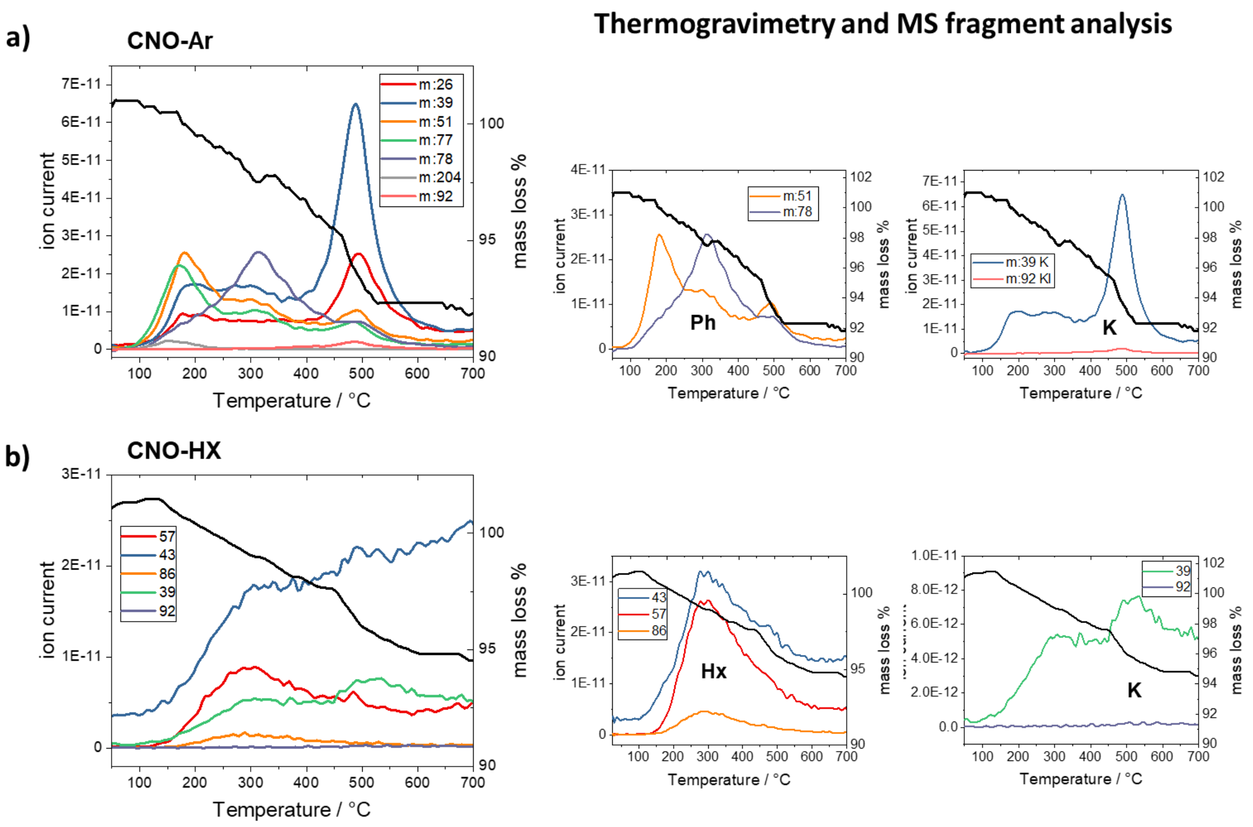

Figure S8: Mass loss/temperature graph from TGA analysis combined to mass analysis. The injected gas was analyzed directly from TGA, showing the development of the main m/z peaks, mainly localized at 300 °C for the covalently attached organic moieties (a) Aryl-CNO and b) Hexyl-CNO) and at 500°C for the residual potassium trapped inside the CNOs.

FTIR was performed on ZnSe plate. The CNO-aryl derivative shows stronger bands at 2920, 2852 and 1675 cm^{-1} than the pristine-CNO which correspond to the aromatic C-H stretching (st) and C-C st, respectively. New bands appear at 1560 cm^{-1} for aromatic C-C st and 670 cm^{-1} which is characteristic for the aromatic C-H bending out of plane, thus corroborating the functionalization. UV-vis absorption studies were performed in NMP as it has been addressed that is the solvent showing stronger interactions with the outer shell of the nano onions and therefore affords the more stable dispersions.[3] Absorption spectra of the functionalized-CNO, both with hexyl and aryl moieties correlate well to others previously described in the literature (Figure S13b).[3,4]

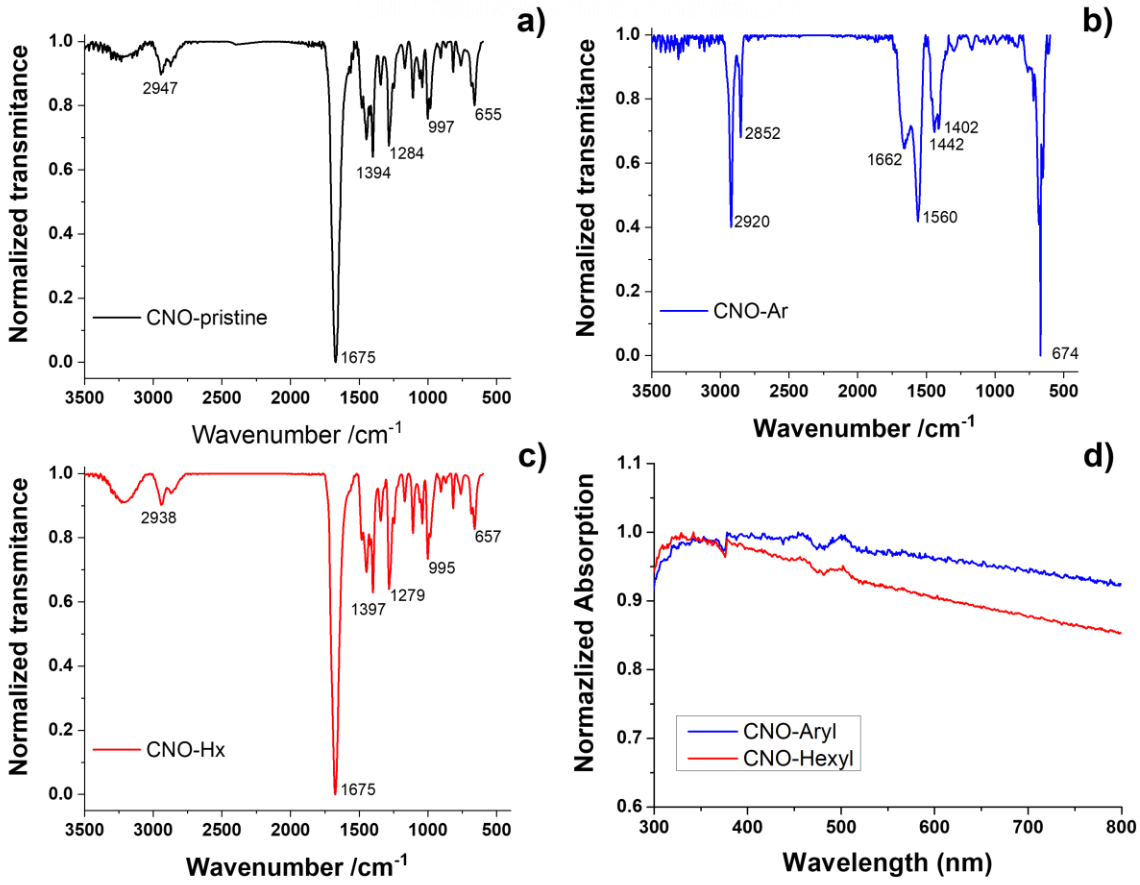

Figure S9: FTIR-ATR analysis of a) pristine CNOs, b) CNO-Aryl and c) CNO-Hexyl; d) UV-vis analysis on NMP at 0.2 mg/mL concentration of CNO-Aryl (blue line) and CNO-Hexyl (red line).

Dynamic Light Scattering experiments (Figure S16) were carried out in order to evaluate the solvated diameter of the CNO and to study the influence of the functionalization on their solubility. The number weighted size distribution in NMP reveals a slight shift of the distribution towards smaller sizes, centered at 250 nm, for both alkyl- and aryl- functionalized samples with respect to the pristine onions. These distributions are far from the CNOs´ nominal size of *ca*. 5 nm. For the case of the THF dispersions, the effects are different for each type of functionalization and thus corroborating that the functional group covalently attached really affects the interaction with the solvent and improves their solubility but only up to a certain point and it does not reach the complete disaggregation into individual onions. Accordingly, TEM images show always clumps of several onions which is in good agreement with the work of Kuznetsov *et al*[5] who described the assembly of CNO in aggregates which in principle correspond in size to the original nano-diamond agglomerates.[6,7] This fact has been further explained by the group of Presser who examined the aggregates' size and the resulting porosity caused by the graphitic interparticle connections that have been well establish for the precursor nanodiamonds.[8] Moreover the group of Mücklich carried out a detailed dispersion analysis finding a hydrodynamic radius of 250 nm which corresponds well to our result and reaching a limit in the disruption of the aggregates *via* sonication at around 40–70 nm.[9]

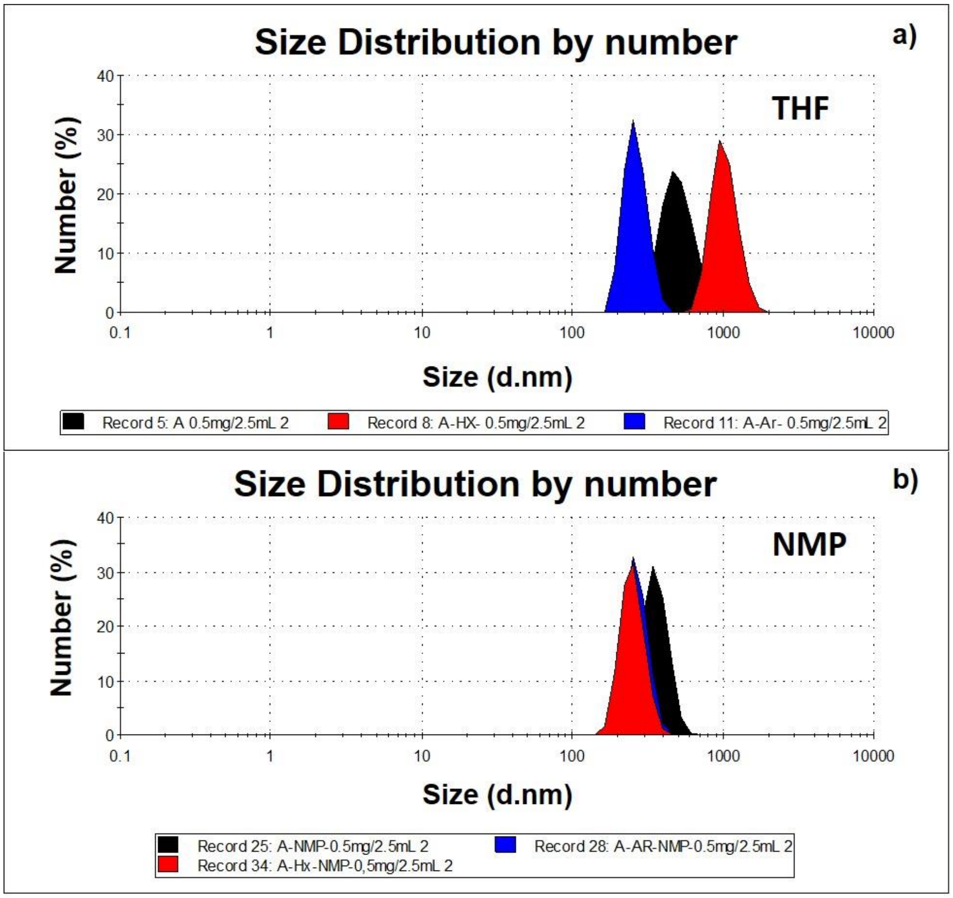

Figure S10: Dynamic light scattering measurement. CNO (0.2 mg/mL) were dispersed into filtered NMP or THF as an alternative solvent for comparison and sonicated for 15 min before measurement. a) THF solution; b) NMP solution.

Statistical analysis of temperature dependent Raman

1.-CNO-Aryl

<table>
<tr><th>T/°C</th><th>Mean (25 spectra)</th><th>D/G</th><th>2D/G</th></tr>
<tr><td>25</td><td>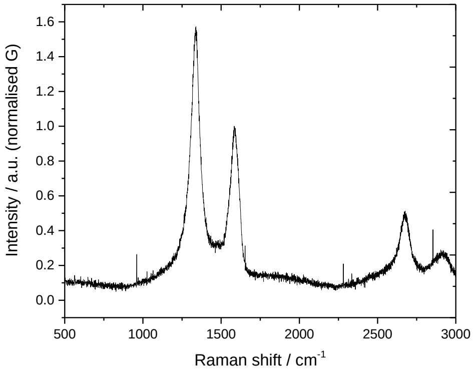
</td><td>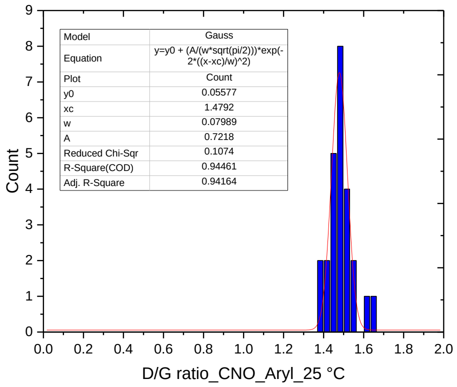
</td><td>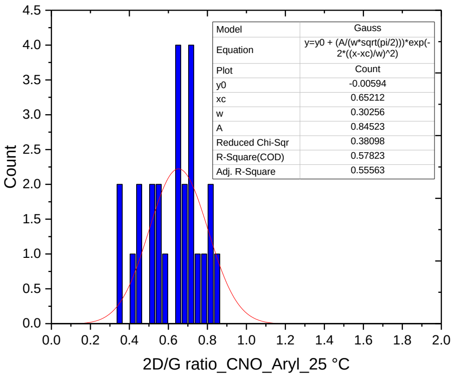
</td></tr>
<tr><td>50</td><td>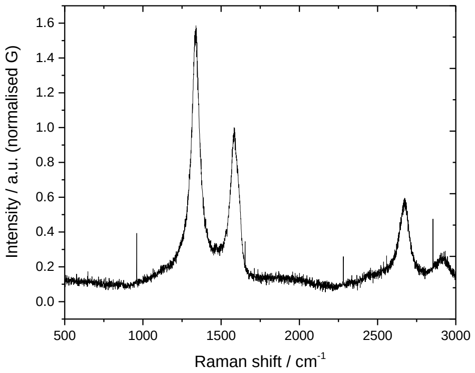
</td><td>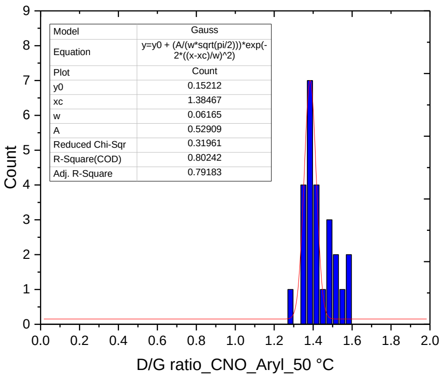
</td><td>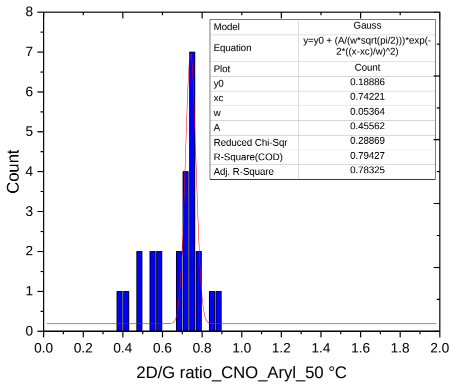
</td></tr>
</table>

<table>
<tr>
<td>75</td>
<td>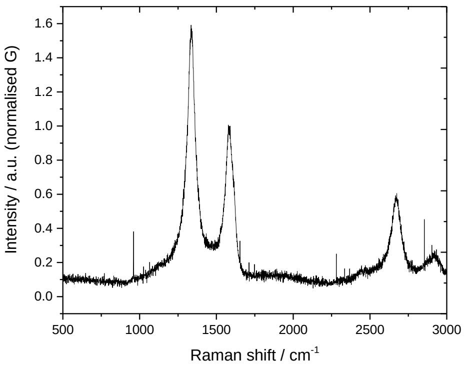
</td>
<td>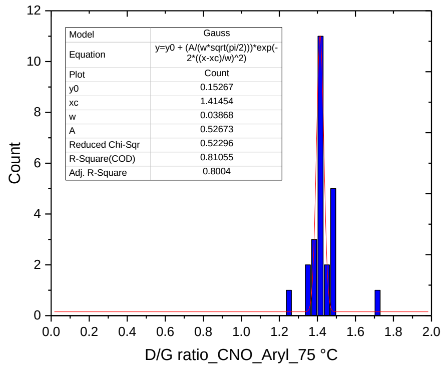
</td>
<td>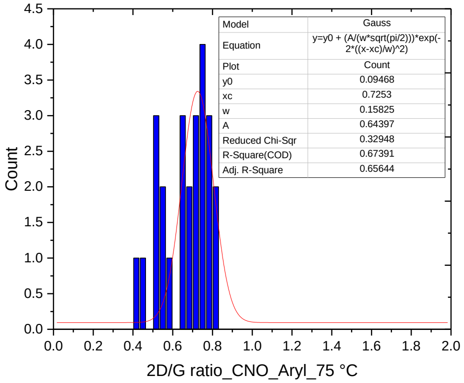
</td>
</tr>
<tr>
<td>100</td>
<td>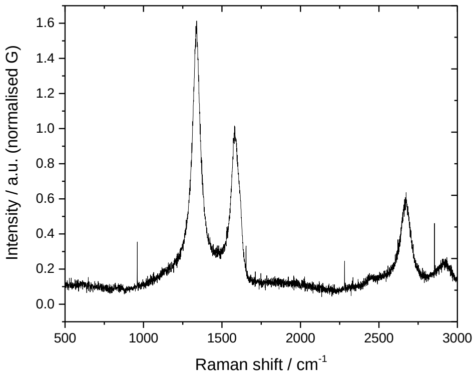
</td>
<td>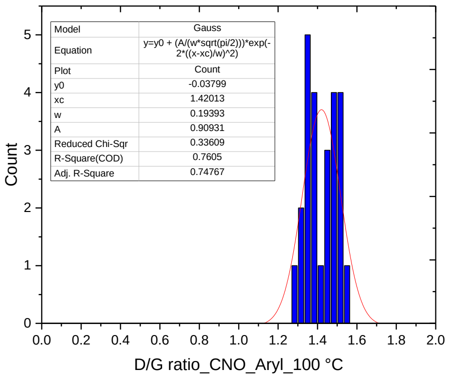
</td>
<td>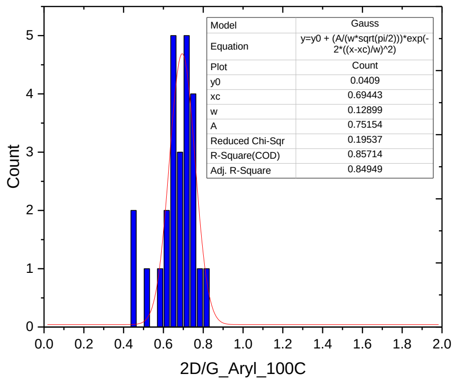
</td>
</tr>
</table>

125

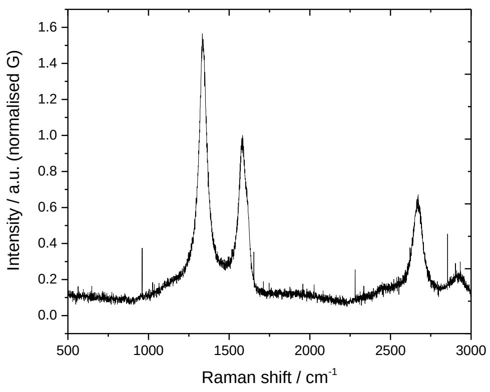

Intensity / a.u. (normalised G)
Raman shift / cm-1

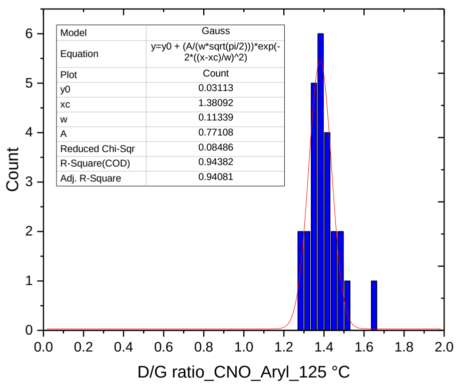

Model Gauss
Equation y=y0 + (A/(w*sqrt(pi/2)))*exp(-2*((x-xc)/w)^2)
Plot Count
y0 0.03113
xc 1.38092
w 0.11339
A 0.77108
Reduced Chi-Sqr 0.08486
R-Square(COD) 0.94382
Adj. R-Square 0.94081
Count
D/G ratio_CNO_Aryl_125 °C

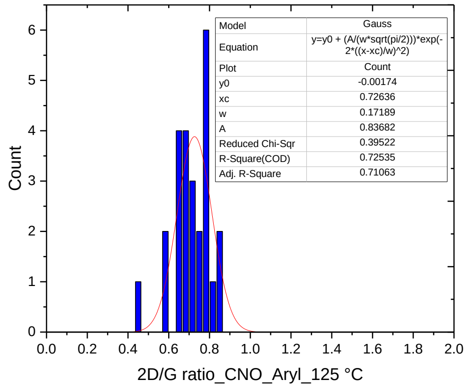

Model Gauss
Equation y=y0 + (A/(w*sqrt(pi/2)))*exp(-2*((x-xc)/w)^2)
Plot Count
y0 -0.00174
xc 0.72636
w 0.17189
A 0.83682
Reduced Chi-Sqr 0.39522
R-Square(COD) 0.72535
Adj. R-Square 0.71063
Count
2D/G ratio_CNO_Aryl_125 °C

150

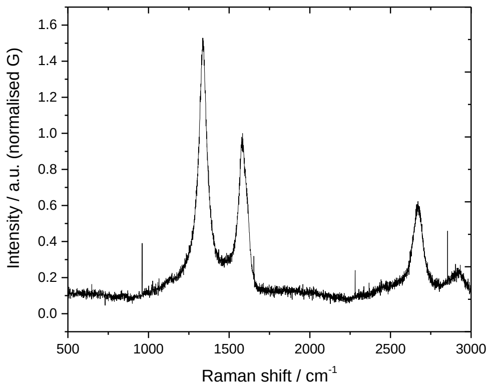

Intensity / a.u. (normalised G)
Raman shift / cm-1

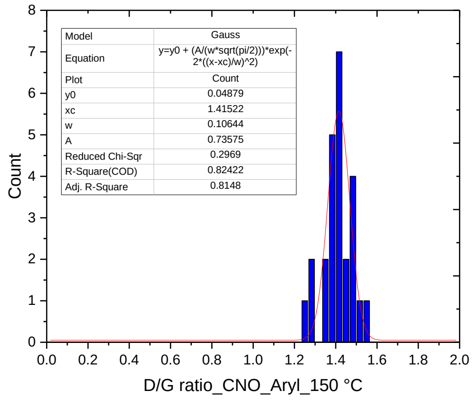

Model Gauss
Equation y=y0 + (A/(w*sqrt(pi/2)))*exp(-2*((x-xc)/w)^2)
Plot Count
y0 0.04879
xc 1.41522
w 0.10644
A 0.73575
Reduced Chi-Sqr 0.2969
R-Square(COD) 0.82422
Adj. R-Square 0.8148
Count
D/G ratio_CNO_Aryl_150 °C

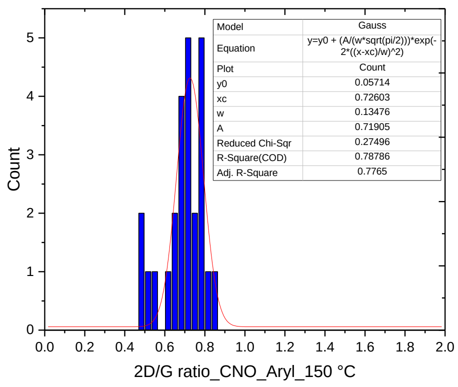

Model Gauss
Equation y=y0 + (A/(w*sqrt(pi/2)))*exp(-2*((x-xc)/w)^2)
Plot Count
y0 0.05714
xc 0.72603
w 0.13476
A 0.71905
Reduced Chi-Sqr 0.27496
R-Square(COD) 0.78786
Adj. R-Square 0.7765
Count
2D/G ratio_CNO_Aryl_150 °C

<table>
<tr><td>175</td><td>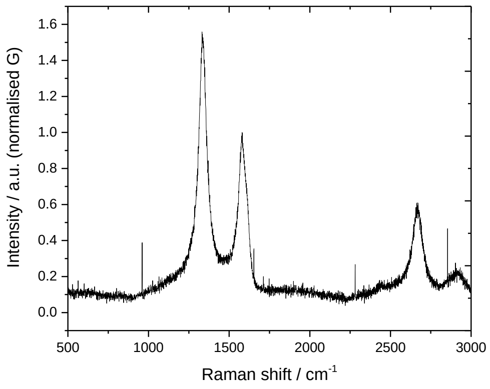
</td><td>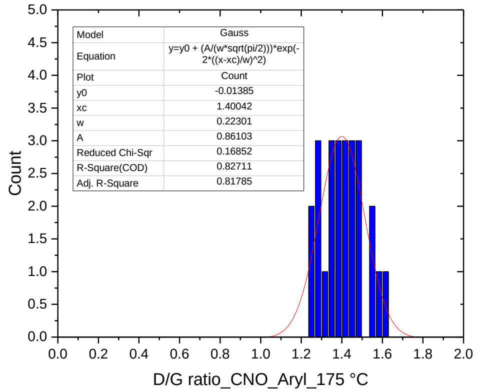
</td><td>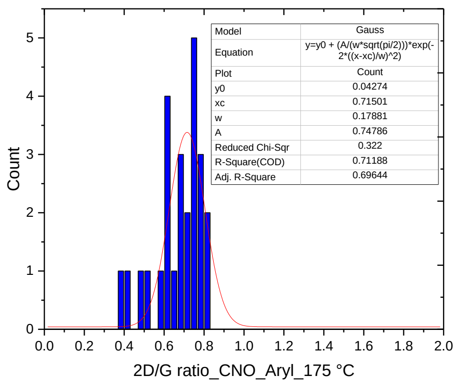
</td></tr>
<tr><td>200</td><td>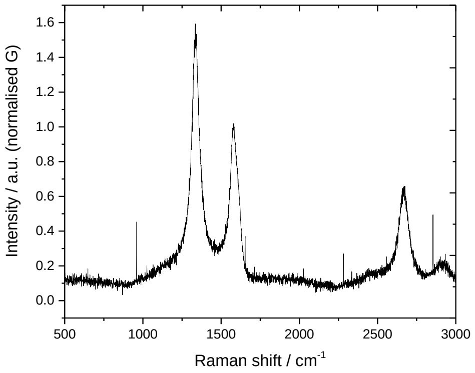
</td><td>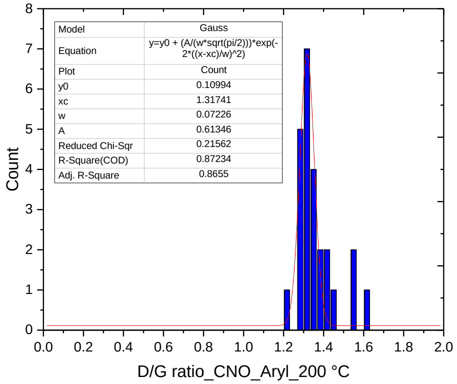
</td><td>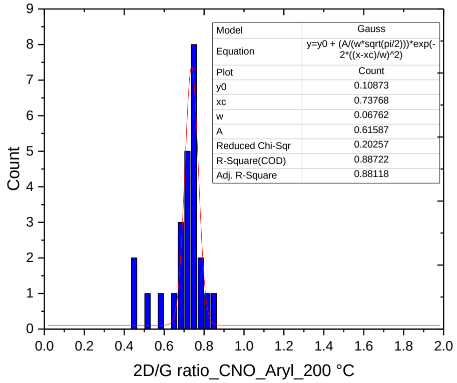
</td></tr>
</table>

225	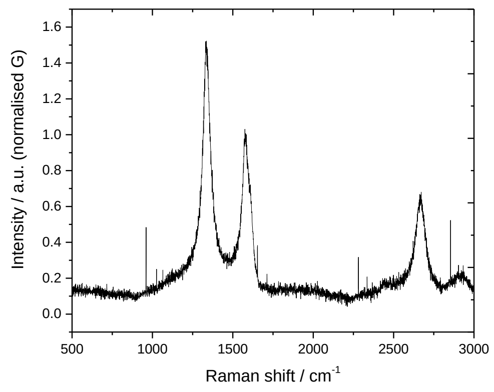 Intensity / a.u. (normalised G)<br>Raman shift / cm-1	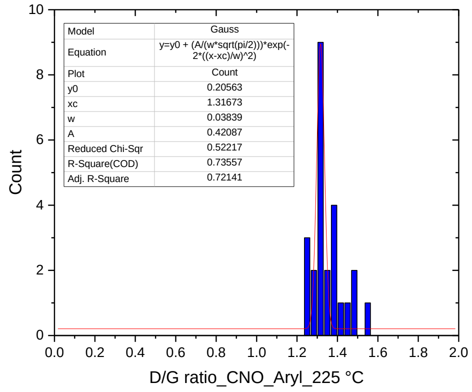 Model Gauss<br>Equation y=y0 + (A/(w*sqrt(pi/2)))*exp(-2*((x-xc)/w)^2)<br>Plot Count<br>y0 0.20563<br>xc 1.31673<br>w 0.03839<br>A 0.42087<br>Reduced Chi-Sqr 0.52217<br>R-Square(COD) 0.73557<br>Adj. R-Square 0.72141<br>Count<br>D/G ratio_CNO_Aryl_225 °C	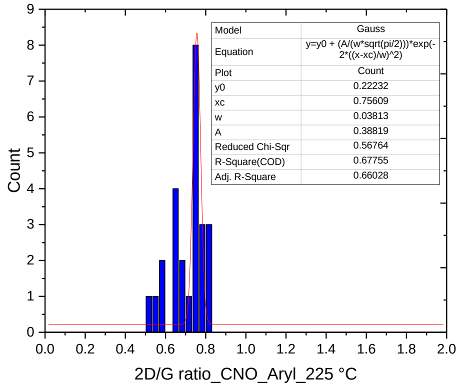 Model Gauss<br>Equation y=y0 + (A/(w*sqrt(pi/2)))*exp(-2*((x-xc)/w)^2)<br>Plot Count<br>y0 0.22232<br>xc 0.75609<br>w 0.03813<br>A 0.38819<br>Reduced Chi-Sqr 0.56764<br>R-Square(COD) 0.67755<br>Adj. R-Square 0.66028<br>Count<br>2D/G ratio_CNO_Aryl_225 °C
250	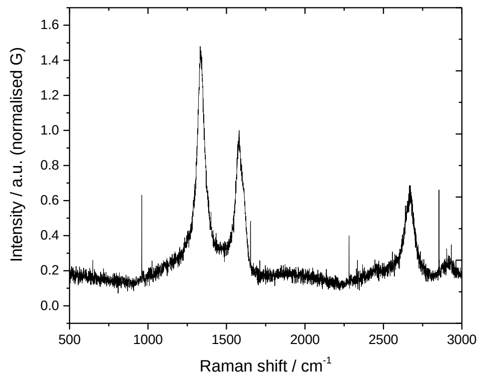 Intensity / a.u. (normalised G)<br>Raman shift / cm-1	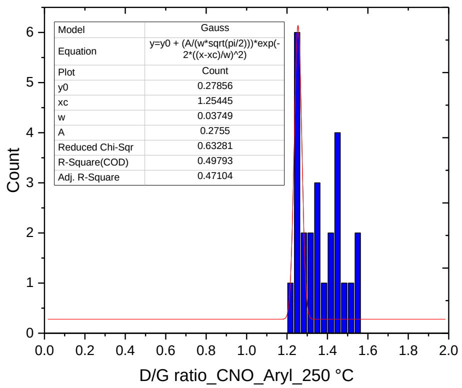 Model Gauss<br>Equation y=y0 + (A/(w*sqrt(pi/2)))*exp(-2*((x-xc)/w)^2)<br>Plot Count<br>y0 0.27856<br>xc 1.25445<br>w 0.03749<br>A 0.2755<br>Reduced Chi-Sqr 0.63281<br>R-Square(COD) 0.49793<br>Adj. R-Square 0.47104<br>Count<br>D/G ratio_CNO_Aryl_250 °C	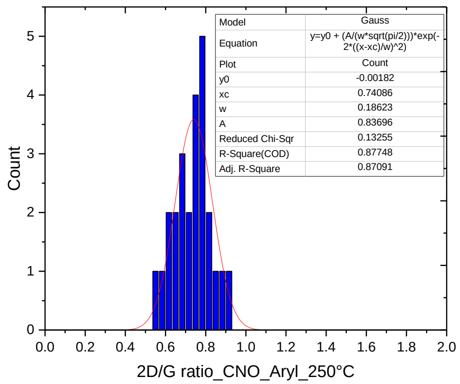 Model Gauss<br>Equation y=y0 + (A/(w*sqrt(pi/2)))*exp(-2*((x-xc)/w)^2)<br>Plot Count<br>y0 -0.00182<br>xc 0.74086<br>w 0.18623<br>A 0.83696<br>Reduced Chi-Sqr 0.13255<br>R-Square(COD) 0.87748<br>Adj. R-Square 0.87091<br>Count<br>2D/G ratio_CNO_Aryl_250°C

275

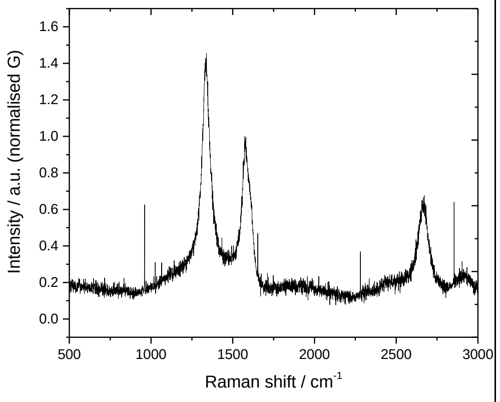

Intensity / a.u. (normalised G)
Raman shift / cm-1

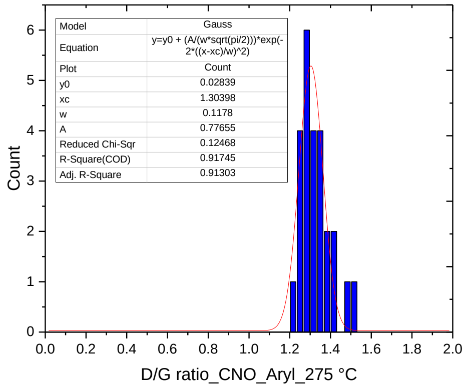

Model Gauss
Equation y=y0 + (A/(w*sqrt(pi/2)))*exp(-2*((x-xc)/w)^2)
Plot Count
y0 0.02839
xc 1.30398
w 0.1178
A 0.77655
Reduced Chi-Sqr 0.12468
R-Square(COD) 0.91745
Adj. R-Square 0.91303
Count
D/G ratio_CNO_Aryl_275 °C

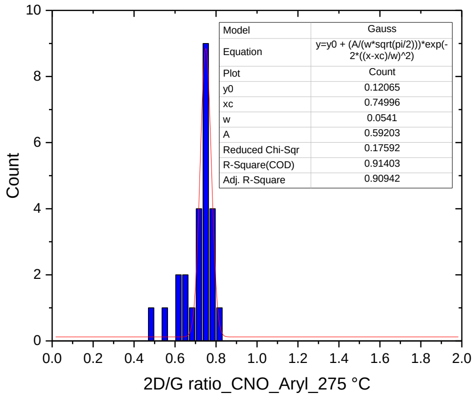

Model Gauss
Equation y=y0 + (A/(w*sqrt(pi/2)))*exp(-2*((x-xc)/w)^2)
Plot Count
y0 0.12065
xc 0.74996
w 0.0541
A 0.59203
Reduced Chi-Sqr 0.17592
R-Square(COD) 0.91403
Adj. R-Square 0.90942
Count
2D/G ratio_CNO_Aryl_275 °C

300

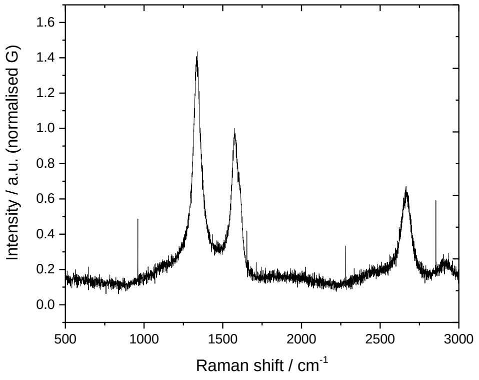

Intensity / a.u. (normalised G)
Raman shift / cm-1

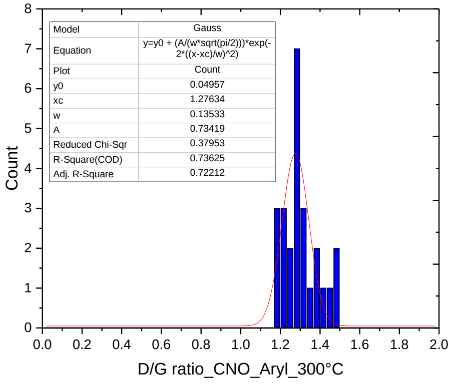

Model Gauss
Equation y=y0 + (A/(w*sqrt(pi/2)))*exp(-2*((x-xc)/w)^2)
Plot Count
y0 0.04957
xc 1.27634
w 0.13533
A 0.73419
Reduced Chi-Sqr 0.37953
R-Square(COD) 0.73625
Adj. R-Square 0.72212
Count
D/G ratio_CNO_Aryl_300°C

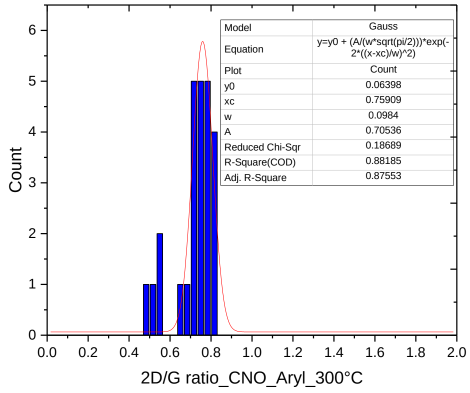

Model Gauss
Equation y=y0 + (A/(w*sqrt(pi/2)))*exp(-2*((x-xc)/w)^2)
Plot Count
y0 0.06398
xc 0.75909
w 0.0984
A 0.70536
Reduced Chi-Sqr 0.18689
R-Square(COD) 0.88185
Adj. R-Square 0.87553
Count
2D/G ratio_CNO_Aryl_300°C

325	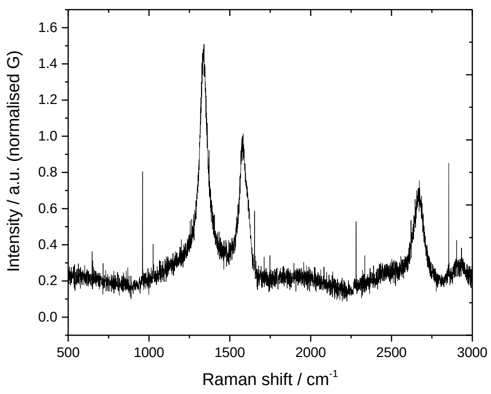 Intensity / a.u. (normalised G)<br>Raman shift / cm^{-1}	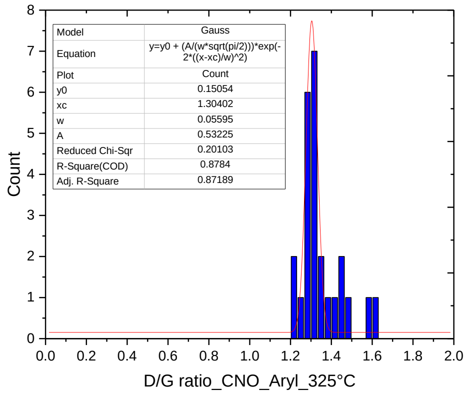 Model Gauss<br>Equation y=y0 + (A/(w*sqrt(pi/2)))*exp(-2*((x-xc)/w)^2)<br>Plot Count<br>y0 0.15054<br>xc 1.30402<br>w 0.05595<br>A 0.53225<br>Reduced Chi-Sqr 0.20103<br>R-Square(COD) 0.8784<br>Adj. R-Square 0.87189<br>Count<br>D/G ratio_CNO_Aryl_325°C	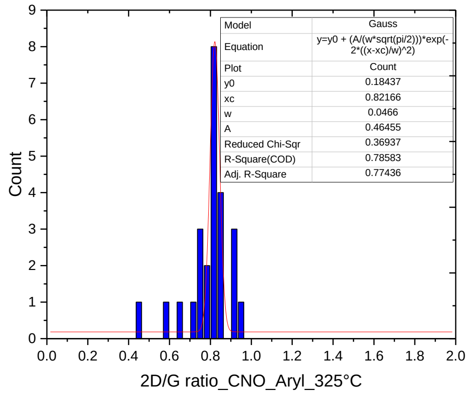 Model Gauss<br>Equation y=y0 + (A/(w*sqrt(pi/2)))*exp(-2*((x-xc)/w)^2)<br>Plot Count<br>y0 0.18437<br>xc 0.82166<br>w 0.0466<br>A 0.46455<br>Reduced Chi-Sqr 0.36937<br>R-Square(COD) 0.78583<br>Adj. R-Square 0.77436<br>Count<br>2D/G ratio_CNO_Aryl_325°C
350	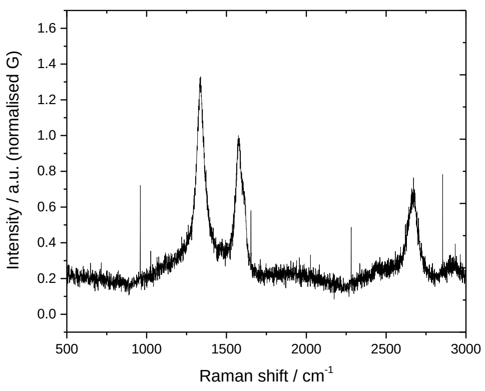 Intensity / a.u. (normalised G)<br>Raman shift / cm^{-1}	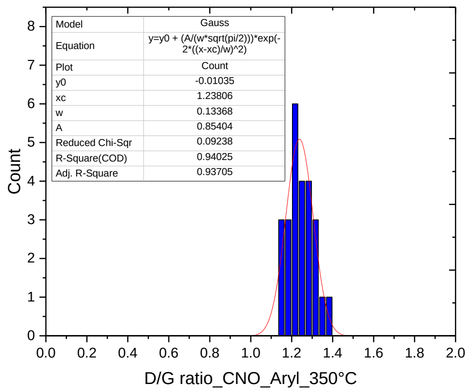 Model Gauss<br>Equation y=y0 + (A/(w*sqrt(pi/2)))*exp(-2*((x-xc)/w)^2)<br>Plot Count<br>y0 -0.01035<br>xc 1.23806<br>w 0.13368<br>A 0.85404<br>Reduced Chi-Sqr 0.09238<br>R-Square(COD) 0.94025<br>Adj. R-Square 0.93705<br>Count<br>D/G ratio_CNO_Aryl_350°C	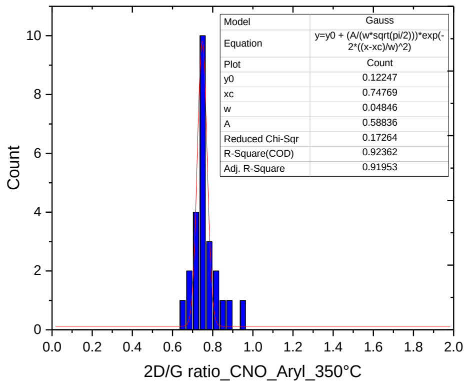 Model Gauss<br>Equation y=y0 + (A/(w*sqrt(pi/2)))*exp(-2*((x-xc)/w)^2)<br>Plot Count<br>y0 0.12247<br>xc 0.74769<br>w 0.04846<br>A 0.58836<br>Reduced Chi-Sqr 0.17264<br>R-Square(COD) 0.92362<br>Adj. R-Square 0.91953<br>Count<br>2D/G ratio_CNO_Aryl_350°C

<table>
<tr><td>400</td><td>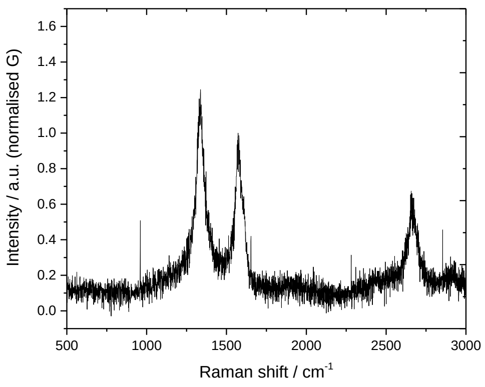
</td><td>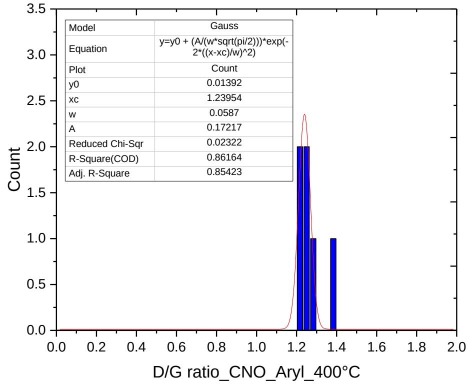
</td><td>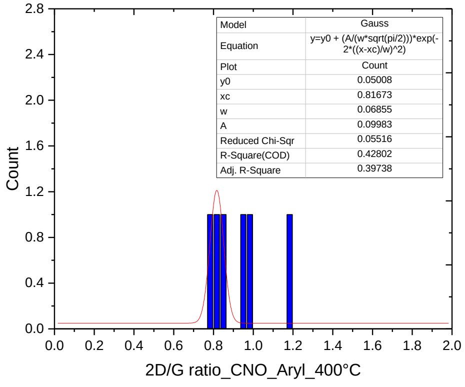
</td></tr>
<tr><td></td><td></td><td></td><td></td></tr>
</table>

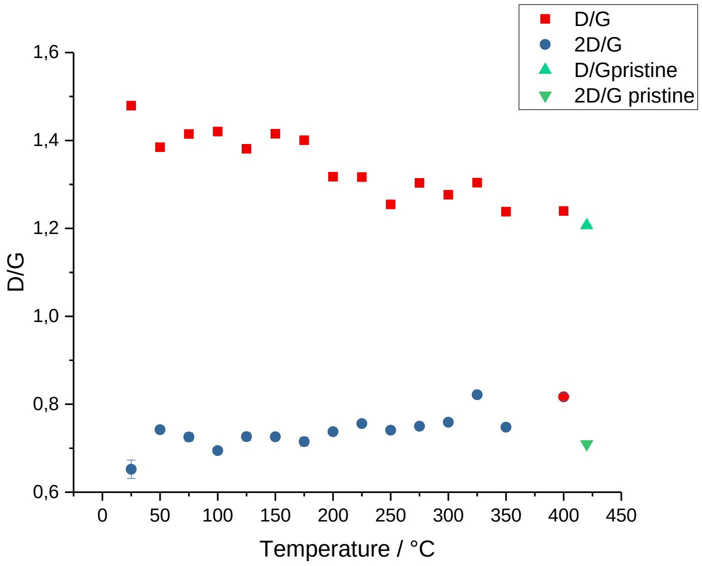

2.-CNO-Hexyl

<table>
<tr><th>T/°C</th><th></th><th>D/G</th><th>2D/G</th></tr>
<tr><td>25</td>
<td>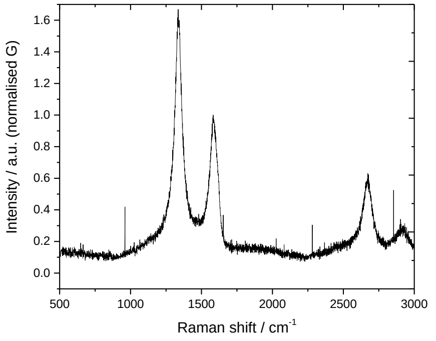</td>
<td>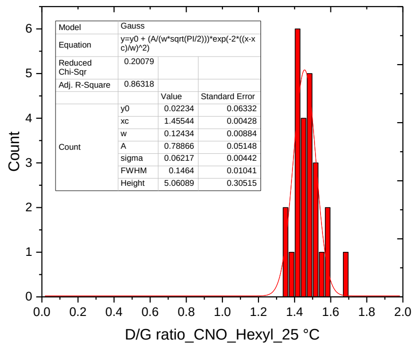</td>
<td>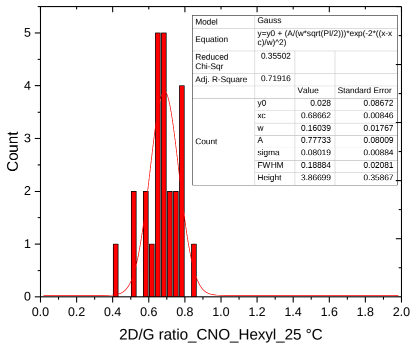</td></tr>
<tr><td>50</td>
<td>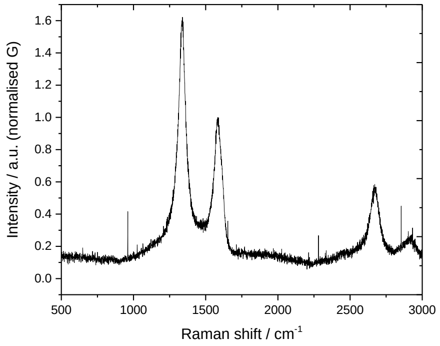</td>
<td>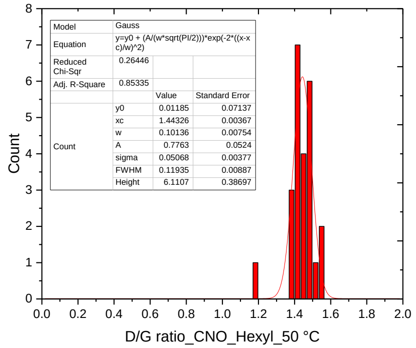</td>
<td>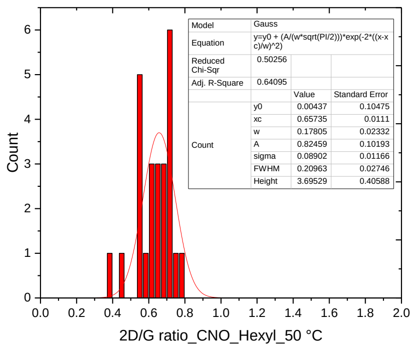</td></tr>
</table>

75	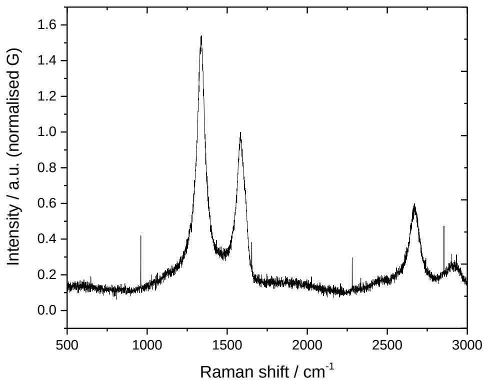Intensity / a.u. (normalised G)<br>Raman shift / cm-1	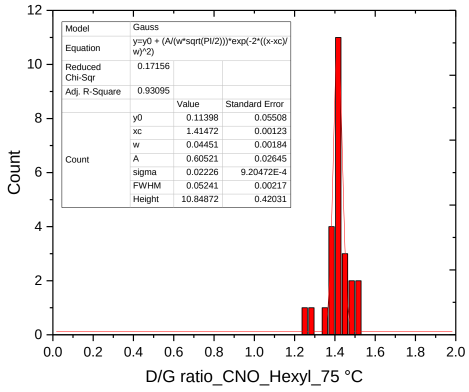Model Gauss<br>Equation y=y0 + (A/(w*sqrt(PI/2)))*exp(-2*((x-xc)/w)^2)<br>Reduced Chi-Sqr 0.17156<br>Adj. R-Square 0.93095<br>Value Standard Error<br>Count y0 0.11398 0.05508<br>xc 1.41472 0.00123<br>w 0.04451 0.00184<br>A 0.60521 0.02645<br>sigma 0.02226 9.20472E-4<br>FWHM 0.05241 0.00217<br>Height 10.84872 0.42031<br>Count<br>D/G ratio_CNO_Hexyl_75 °C	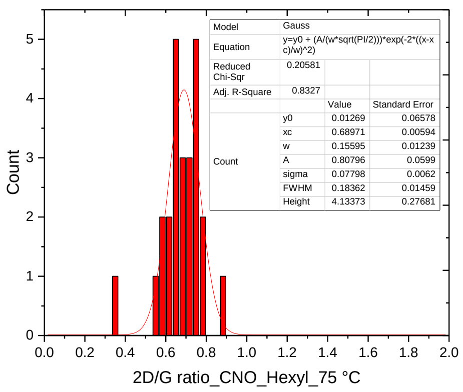Model Gauss<br>Equation y=y0 + (A/(w*sqrt(PI/2)))*exp(-2*((x-xc)/w)^2)<br>Reduced Chi-Sqr 0.20581<br>Adj. R-Square 0.8327<br>Value Standard Error<br>Count y0 0.01269 0.06578<br>xc 0.68971 0.00594<br>w 0.15595 0.01239<br>A 0.80796 0.0599<br>sigma 0.07798 0.0062<br>FWHM 0.18362 0.01459<br>Height 4.13373 0.27681<br>Count<br>2D/G ratio_CNO_Hexyl_75 °C
100	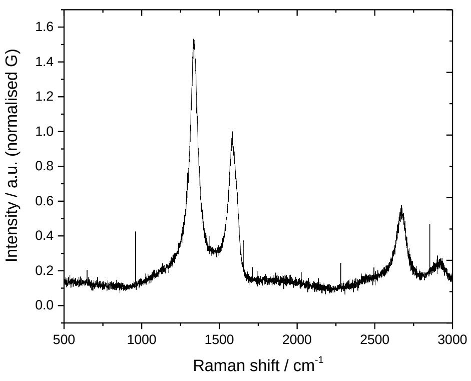Intensity / a.u. (normalised G)<br>Raman shift / cm-1	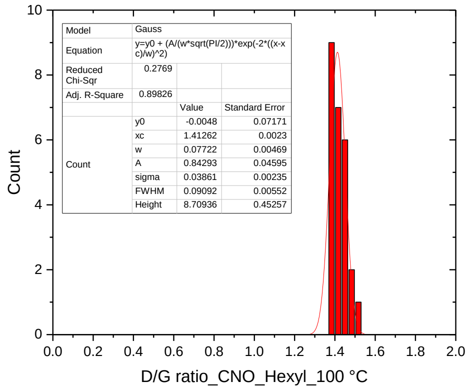Model Gauss<br>Equation y=y0 + (A/(w*sqrt(PI/2)))*exp(-2*((x-xc)/w)^2)<br>Reduced Chi-Sqr 0.2769<br>Adj. R-Square 0.89826<br>Value Standard Error<br>Count y0 -0.0048 0.07171<br>xc 1.41262 0.0023<br>w 0.07722 0.00469<br>A 0.84293 0.04595<br>sigma 0.03861 0.00235<br>FWHM 0.09092 0.00552<br>Height 8.70936 0.45257<br>Count<br>D/G ratio_CNO_Hexyl_100 °C	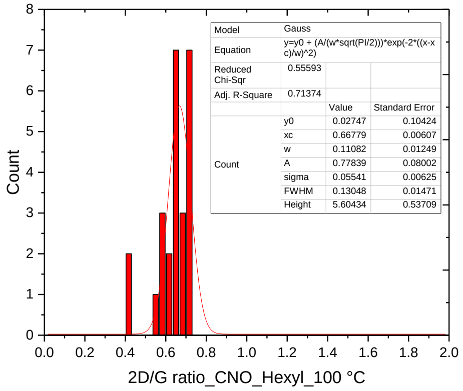Model Gauss<br>Equation y=y0 + (A/(w*sqrt(PI/2)))*exp(-2*((x-xc)/w)^2)<br>Reduced Chi-Sqr 0.55593<br>Adj. R-Square 0.71374<br>Value Standard Error<br>Count y0 0.02747 0.10424<br>xc 0.66779 0.00607<br>w 0.11082 0.01249<br>A 0.77839 0.08002<br>sigma 0.05541 0.00625<br>FWHM 0.13048 0.01471<br>Height 5.60434 0.53709<br>Count<br>2D/G ratio_CNO_Hexyl_100 °C

125

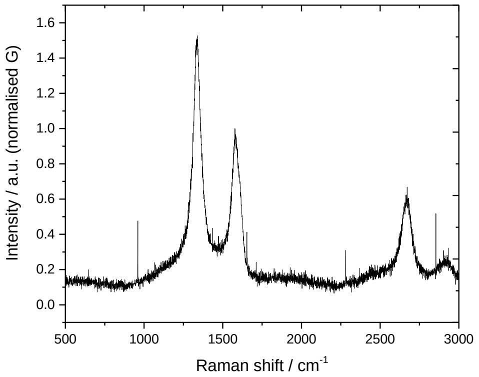
Intensity / a.u. (normalised G)
Raman shift / cm-1

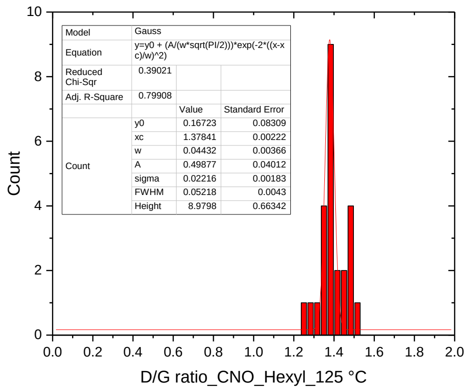
Model Gauss
Equation y=y0 + (A/(w*sqrt(PI/2)))*exp(-2*((x-xc)/w)^2)
Reduced Chi-Sqr 0.39021
Adj. R-Square 0.79908
Value Standard Error
Count y0 0.16723 0.08309
xc 1.37841 0.00222
w 0.04432 0.00366
A 0.49877 0.04012
sigma 0.02216 0.00183
FWHM 0.05218 0.0043
Height 8.9798 0.66342
Count
D/G ratio_CNO_Hexyl_125 °C

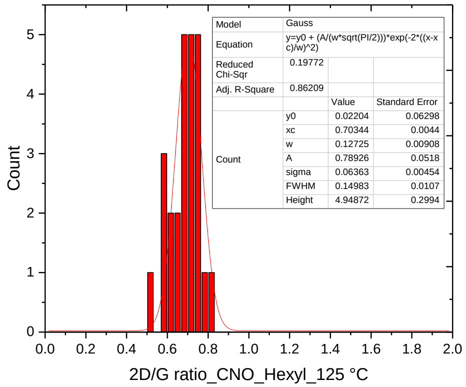
Model Gauss
Equation y=y0 + (A/(w*sqrt(PI/2)))*exp(-2*((x-xc)/w)^2)
Reduced Chi-Sqr 0.19772
Adj. R-Square 0.86209
Value Standard Error
Count y0 0.02204 0.06298
xc 0.70344 0.0044
w 0.12725 0.00908
A 0.78926 0.0518
sigma 0.06363 0.00454
FWHM 0.14983 0.0107
Height 4.94872 0.2994
Count
2D/G ratio_CNO_Hexyl_125 °C

150

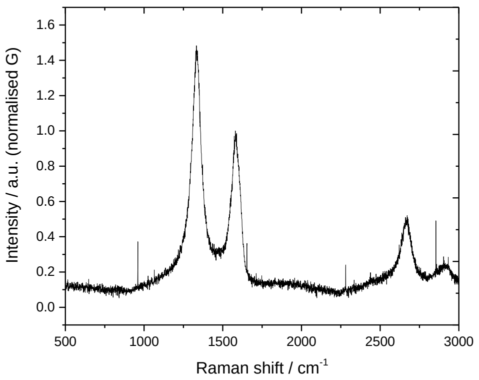
Intensity / a.u. (normalised G)
Raman shift / cm-1

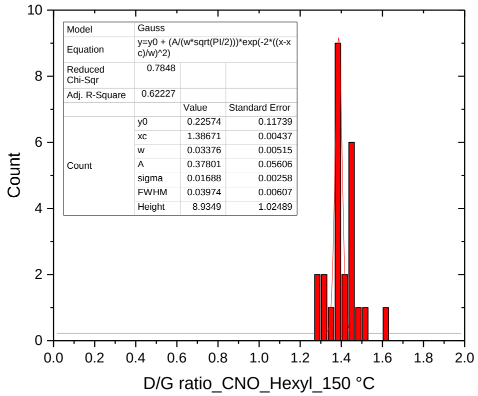
Model Gauss
Equation y=y0 + (A/(w*sqrt(PI/2)))*exp(-2*((x-xc)/w)^2)
Reduced Chi-Sqr 0.7848
Adj. R-Square 0.62227
Value Standard Error
Count y0 0.22574 0.11739
xc 1.38671 0.00437
w 0.03376 0.00515
A 0.37801 0.05606
sigma 0.01688 0.00258
FWHM 0.03974 0.00607
Height 8.9349 1.02489
Count
D/G ratio_CNO_Hexyl_150 °C

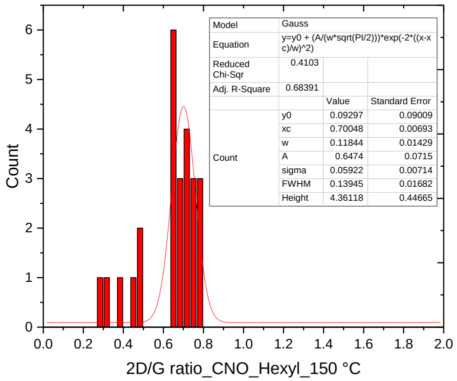
Model Gauss
Equation y=y0 + (A/(w*sqrt(PI/2)))*exp(-2*((x-xc)/w)^2)
Reduced Chi-Sqr 0.4103
Adj. R-Square 0.68391
Value Standard Error
Count y0 0.09297 0.09009
xc 0.70048 0.00693
w 0.11844 0.01429
A 0.6474 0.0715
sigma 0.05922 0.00714
FWHM 0.13945 0.01682
Height 4.36118 0.44665
Count
2D/G ratio_CNO_Hexyl_150 °C

<table>
<tr>
<td>175</td>
<td>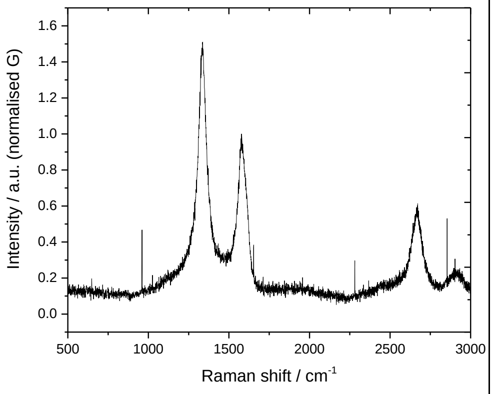
</td>
<td>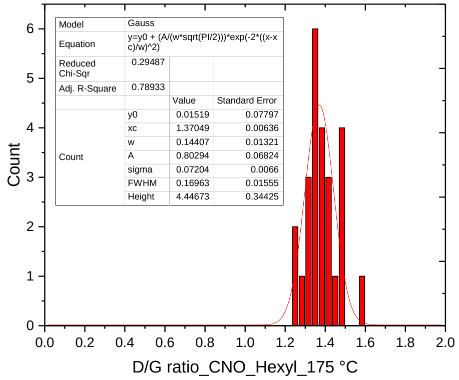
</td>
<td>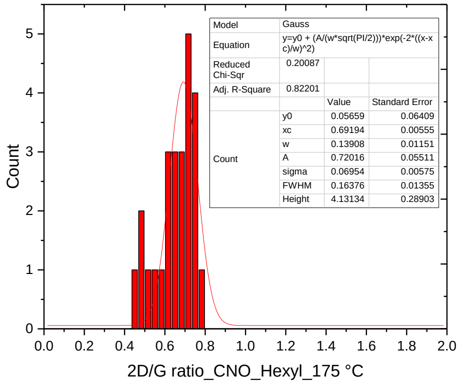
</td>
</tr>
<tr>
<td>200</td>
<td>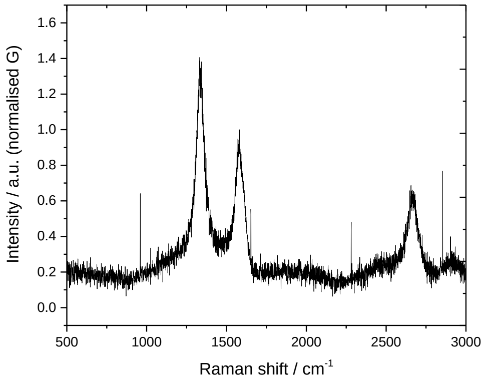
</td>
<td>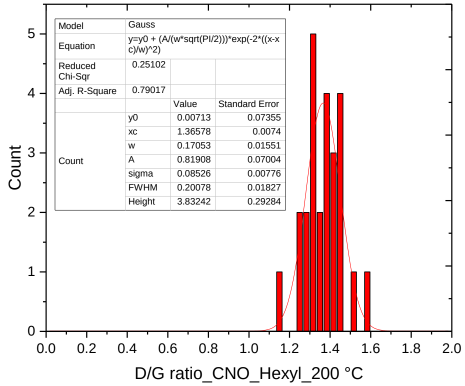
</td>
<td>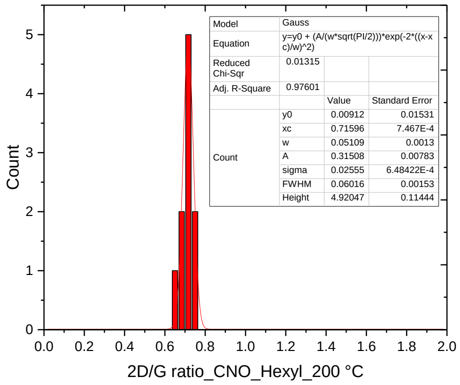
</td>
</tr>
</table>

<table>
<tr>
<td>225</td>
<td>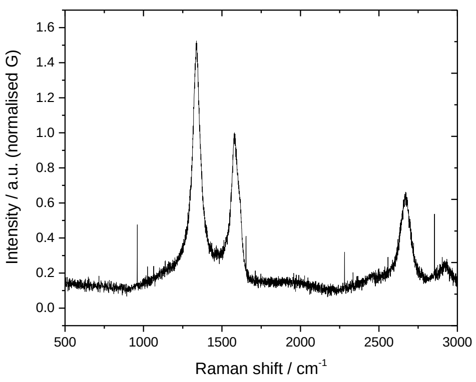
</td>
<td>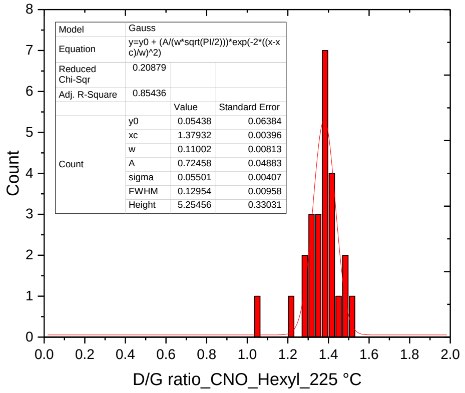
</td>
<td>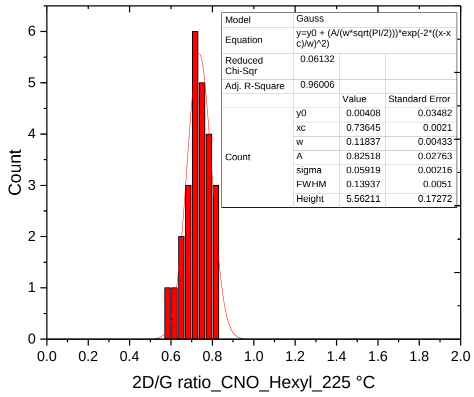
</td>
</tr>
<tr>
<td>250</td>
<td>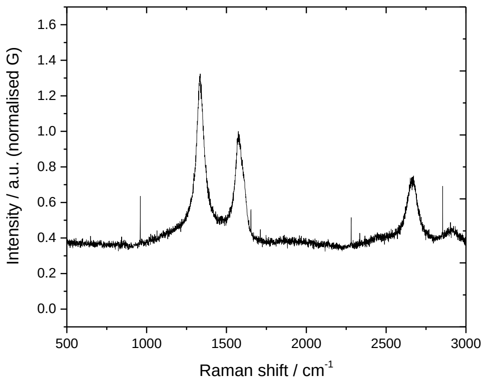
</td>
<td>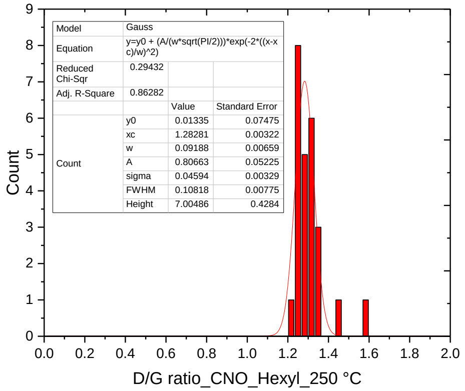
</td>
<td>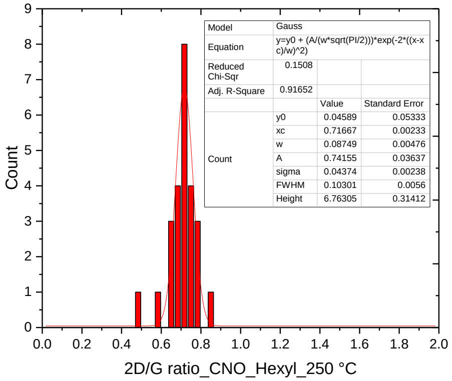
</td>
</tr>
</table>

275

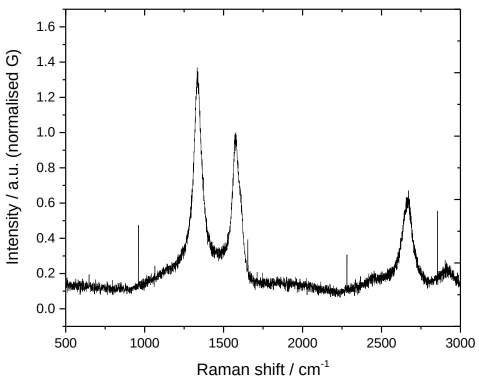
Intensity / a.u. (normalised G)
Raman shift / cm-1

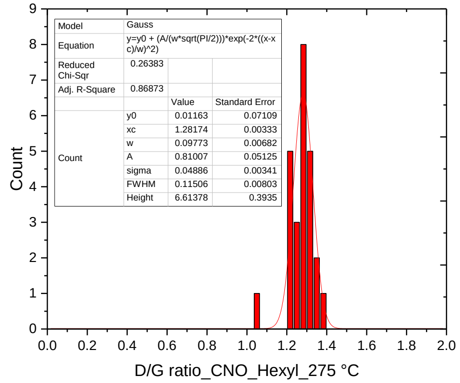
Model | Gauss
Equation | y=y0 + (A/(w*sqrt(PI/2)))*exp(-2*((x-xc)/w)^2)
Reduced Chi-Sqr | 0.26383
Adj. R-Square | 0.86873
Value | Standard Error
Count
y0 | 0.01163 | 0.07109
xc | 1.28174 | 0.00333
w | 0.09773 | 0.00682
A | 0.81007 | 0.05125
sigma | 0.04886 | 0.00341
FWHM | 0.11506 | 0.00803
Height | 6.61378 | 0.3935
Count
D/G ratio_CNO_Hexyl_275 °C

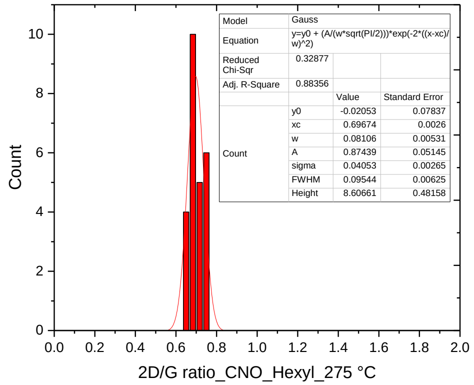
Model | Gauss
Equation | y=y0 + (A/(w*sqrt(PI/2)))*exp(-2*((x-xc)/w)^2)
Reduced Chi-Sqr | 0.32877
Adj. R-Square | 0.88356
Value | Standard Error
Count
y0 | -0.02053 | 0.07837
xc | 0.69674 | 0.0026
w | 0.08106 | 0.00531
A | 0.87439 | 0.05145
sigma | 0.04053 | 0.00265
FWHM | 0.09544 | 0.00625
Height | 8.60661 | 0.48158
Count
2D/G ratio_CNO_Hexyl_275 °C

300

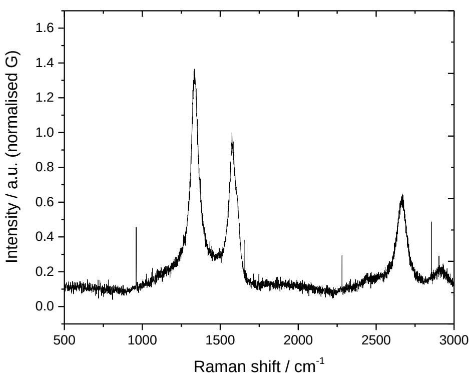
Intensity / a.u. (normalised G)
Raman shift / cm-1

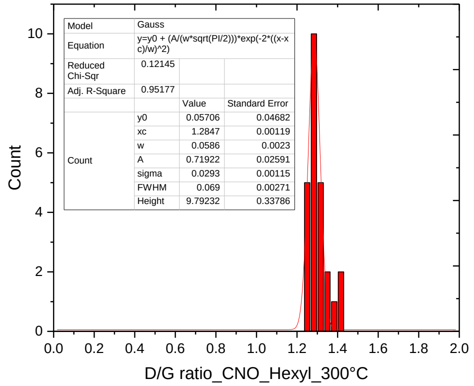
Model | Gauss
Equation | y=y0 + (A/(w*sqrt(PI/2)))*exp(-2*((x-xc)/w)^2)
Reduced Chi-Sqr | 0.12145
Adj. R-Square | 0.95177
Value | Standard Error
Count
y0 | 0.05706 | 0.04682
xc | 1.2847 | 0.00119
w | 0.0586 | 0.0023
A | 0.71922 | 0.02591
sigma | 0.0293 | 0.00115
FWHM | 0.069 | 0.00271
Height | 9.79232 | 0.33786
Count
D/G ratio_CNO_Hexyl_300°C

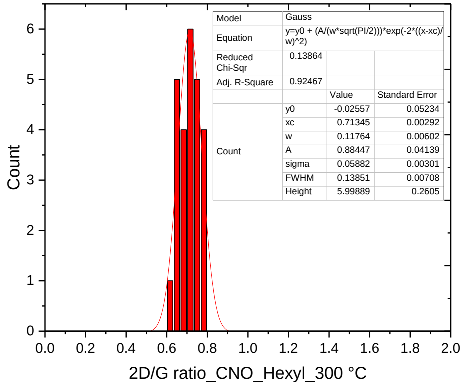
Model | Gauss
Equation | y=y0 + (A/(w*sqrt(PI/2)))*exp(-2*((x-xc)/w)^2)
Reduced Chi-Sqr | 0.13864
Adj. R-Square | 0.92467
Value | Standard Error
Count
y0 | -0.02557 | 0.05234
xc | 0.71345 | 0.00292
w | 0.11764 | 0.00602
A | 0.88447 | 0.04139
sigma | 0.05882 | 0.00301
FWHM | 0.13851 | 0.00708
Height | 5.99889 | 0.2605
Count
2D/G ratio_CNO_Hexyl_300 °C

<table>
<tr>
<td>325</td>
<td>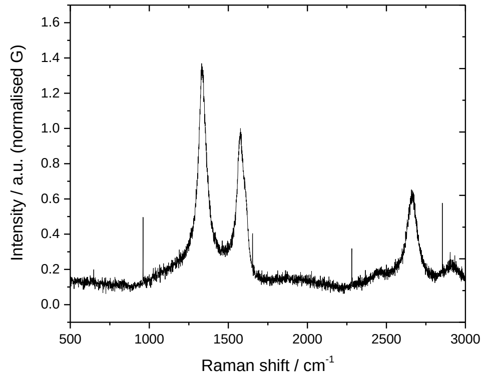
</td>
<td>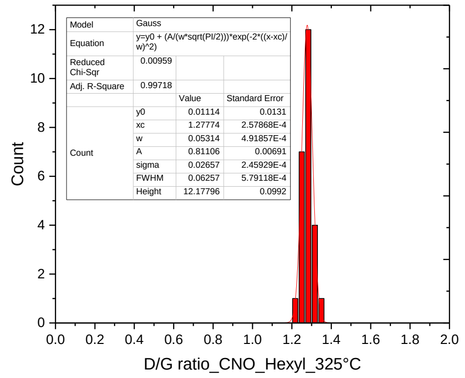
</td>
<td>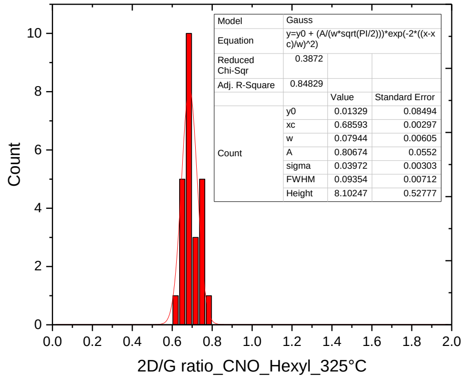
</td>
</tr>
<tr>
<td>350</td>
<td>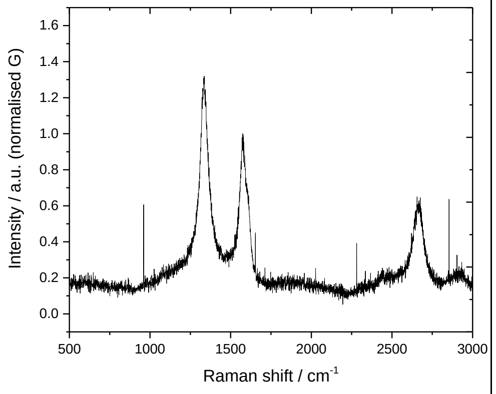
</td>
<td>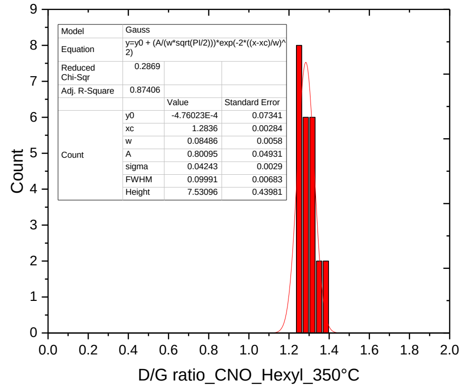
</td>
<td>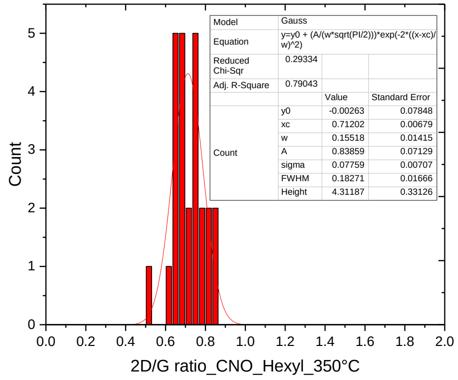
</td>
</tr>
</table>

400

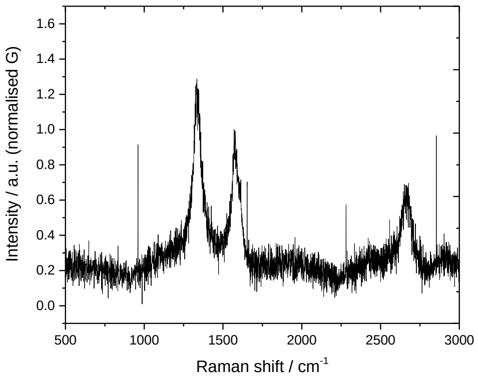

Intensity / a.u. (normalised G)
1.6
1.4
1.2
1.0
0.8
0.6
0.4
0.2
0.0
500
1000
1500
2000
2500
3000
Raman shift / cm-1

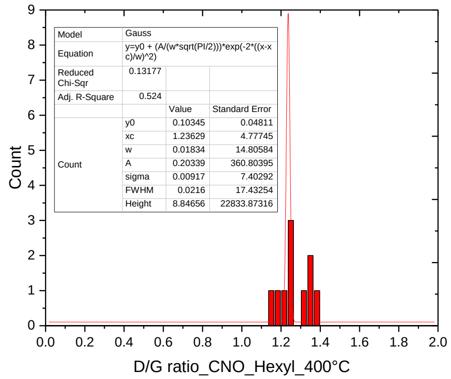

Model | Gauss
Equation | y=y0 + (A/(w*sqrt(PI/2)))*exp(-2*((x-xc)/w)^2)
Reduced Chi-Sqr | 0.13177
Adj. R-Square | 0.524
Value | Standard Error
Count
y0 | 0.10345 | 0.04811
xc | 1.23629 | 4.77745
w | 0.01834 | 14.80584
A | 0.20339 | 360.80395
sigma | 0.00917 | 7.40292
FWHM | 0.0216 | 17.43254
Height | 8.84656 | 22833.87316
Count
D/G ratio_CNO_Hexyl_400°C

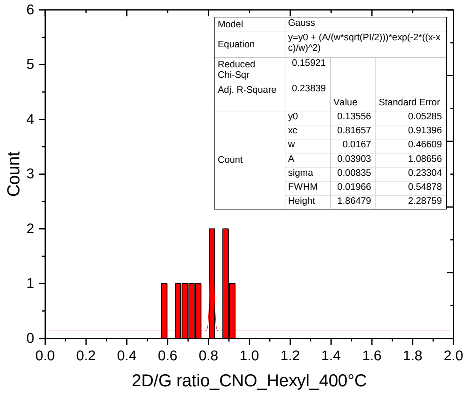

Model | Gauss
Equation | y=y0 + (A/(w*sqrt(PI/2)))*exp(-2*((x-xc)/w)^2)
Reduced Chi-Sqr | 0.15921
Adj. R-Square | 0.23839
Value | Standard Error
Count
y0 | 0.13556 | 0.05285
xc | 0.81657 | 0.91396
w | 0.0167 | 0.46609
A | 0.03903 | 1.08656
sigma | 0.00835 | 0.23304
FWHM | 0.01966 | 0.54878
Height | 1.86479 | 2.28759
Count
2D/G ratio_CNO_Hexyl_400°C

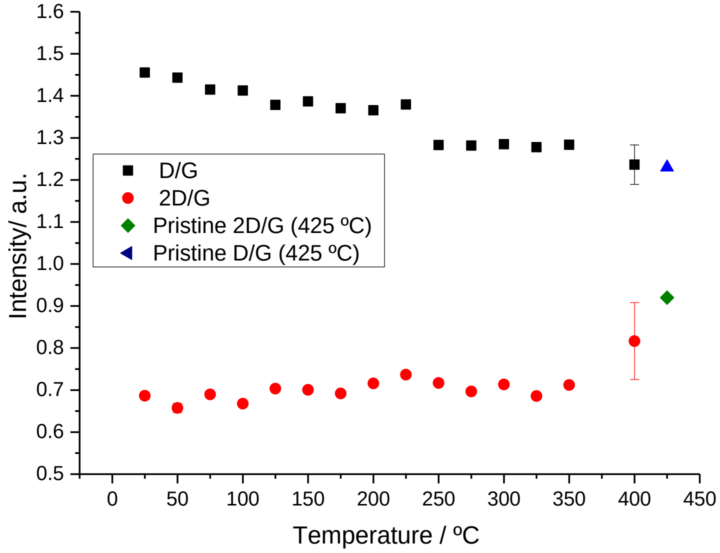

D/G
2D/G
Pristine 2D/G (425 ºC)
Pristine D/G (425 ºC)
Intensity/ a.u.
Temperature / ºC

3.- CNO Pristine

<table>
<tr><th>T/°C</th><th></th><th>D/G</th><th>2D/G</th></tr>
<tr><td>25</td><td>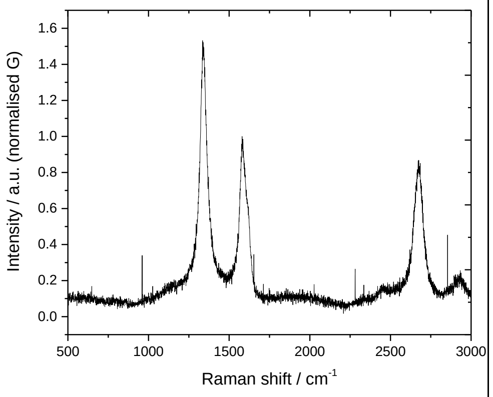
</td><td>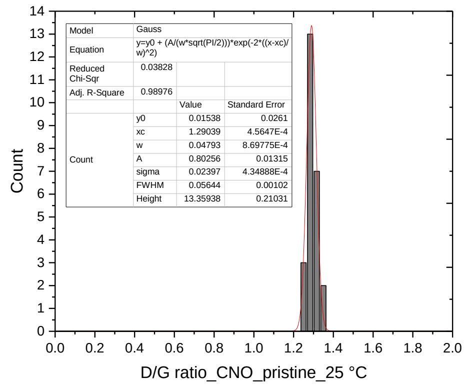
</td><td>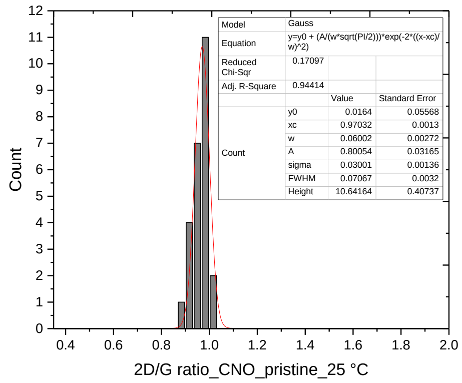
</td></tr>
<tr><td>75</td><td>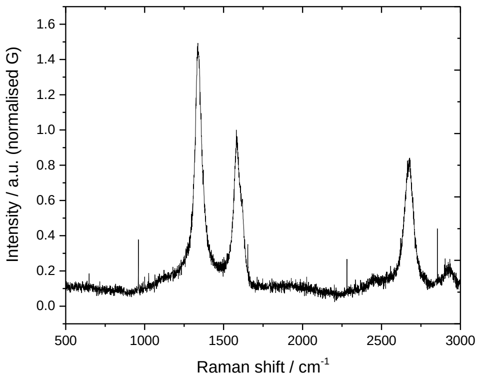
</td><td>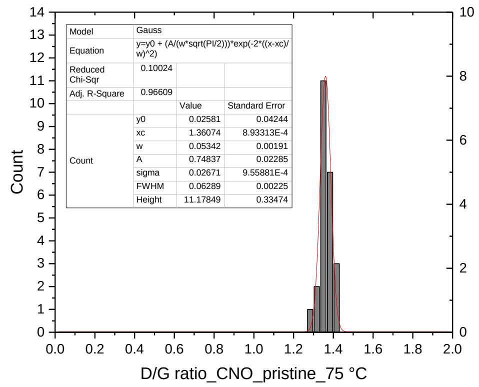
</td><td>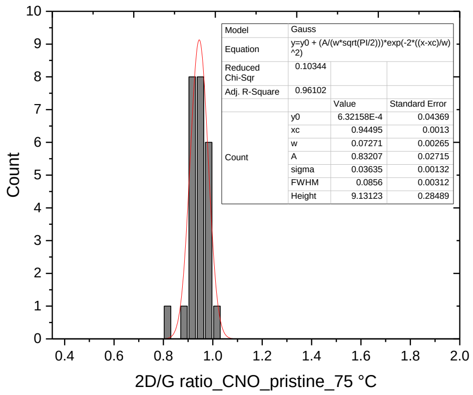
</td></tr>
</table>

125

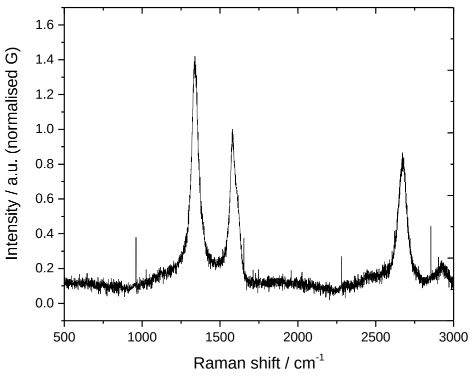

Intensity / a.u. (normalised G)
Raman shift / cm-1

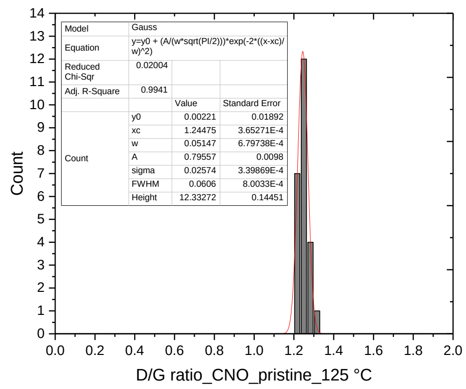

Model Gauss
Equation y=y0 + (A/(w*sqrt(PI/2)))*exp(-2*((x-xc)/w)^2)
Reduced Chi-Sqr 0.02004
Adj. R-Square 0.9941
Value Standard Error
Count
y0 0.00221 0.01892
xc 1.24475 3.65271E-4
w 0.05147 6.79738E-4
A 0.79557 0.0098
sigma 0.02574 3.39869E-4
FWHM 0.0606 8.0033E-4
Height 12.33272 0.14451
Count
D/G ratio_CNO_pristine_125 °C

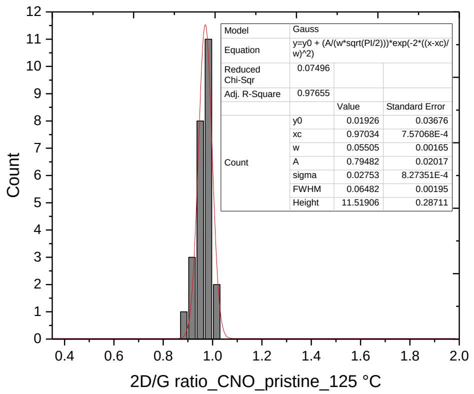

Model Gauss
Equation y=y0 + (A/(w*sqrt(PI/2)))*exp(-2*((x-xc)/w)^2)
Reduced Chi-Sqr 0.07496
Adj. R-Square 0.97655
Value Standard Error
Count
y0 0.01926 0.03676
xc 0.97034 7.57068E-4
w 0.05505 0.00165
A 0.79482 0.02017
sigma 0.02753 8.27351E-4
FWHM 0.06482 0.00195
Height 11.51906 0.28711
Count
2D/G ratio_CNO_pristine_125 °C

175

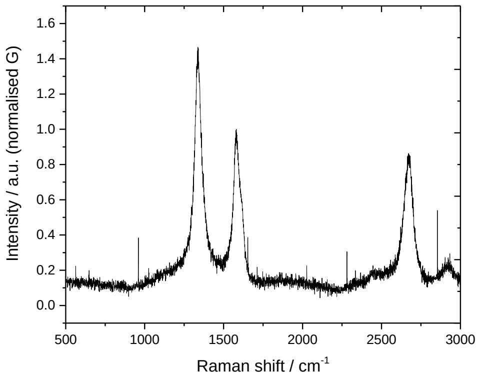

Intensity / a.u. (normalised G)
Raman shift / cm-1

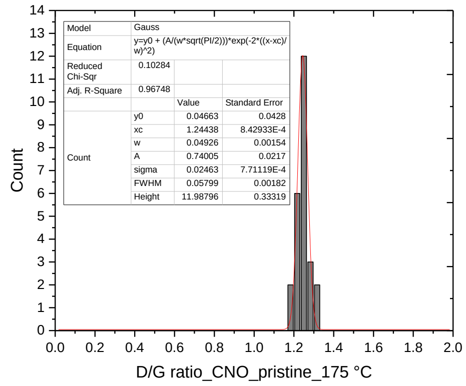

Model Gauss
Equation y=y0 + (A/(w*sqrt(PI/2)))*exp(-2*((x-xc)/w)^2)
Reduced Chi-Sqr 0.10284
Adj. R-Square 0.96748
Value Standard Error
Count
y0 0.04663 0.0428
xc 1.24438 8.42933E-4
w 0.04926 0.00154
A 0.74005 0.0217
sigma 0.02463 7.71119E-4
FWHM 0.05799 0.00182
Height 11.98796 0.33319
Count
D/G ratio_CNO_pristine_175 °C

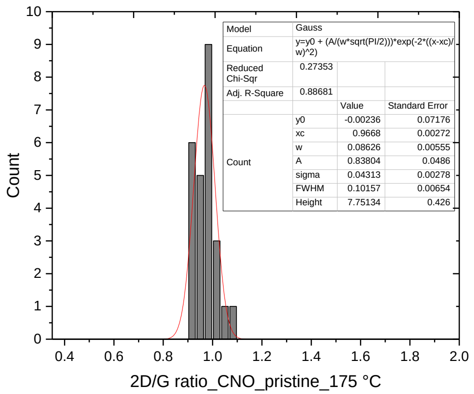

Model Gauss
Equation y=y0 + (A/(w*sqrt(PI/2)))*exp(-2*((x-xc)/w)^2)
Reduced Chi-Sqr 0.27353
Adj. R-Square 0.88681
Value Standard Error
Count
y0 -0.00236 0.07176
xc 0.9668 0.00272
w 0.08626 0.00555
A 0.83804 0.0486
sigma 0.04313 0.00278
FWHM 0.10157 0.00654
Height 7.75134 0.426
Count
2D/G ratio_CNO_pristine_175 °C

<table>
<tr>
<td>225</td>
<td>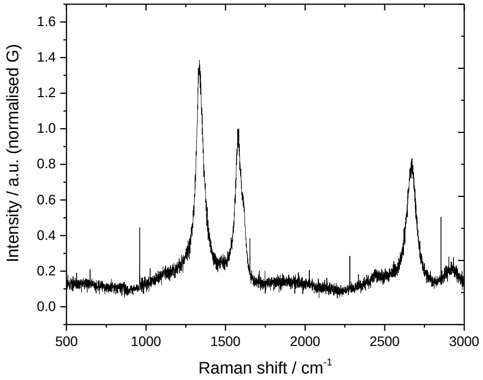
</td>
<td>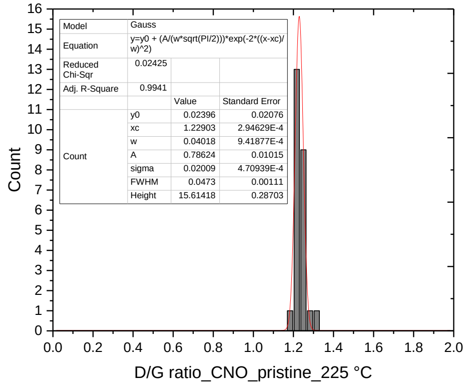
</td>
<td>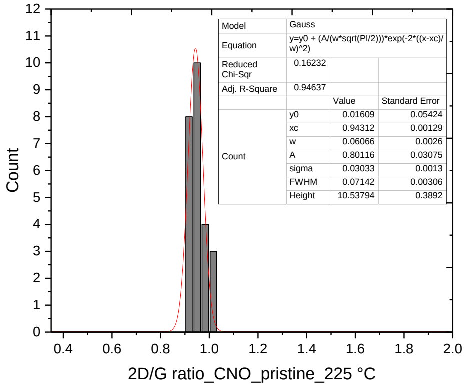
</td>
</tr>
<tr>
<td>275</td>
<td>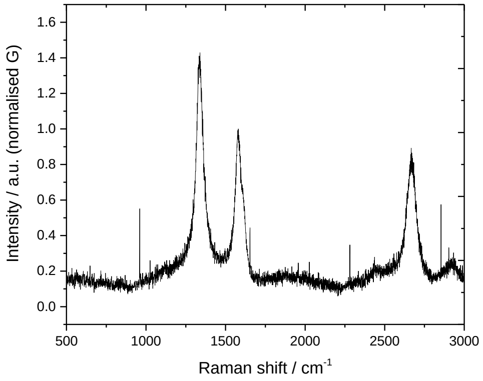
</td>
<td>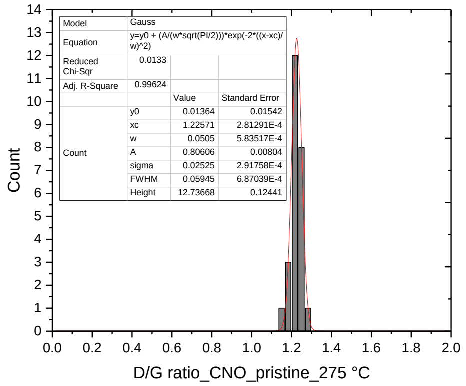
</td>
<td>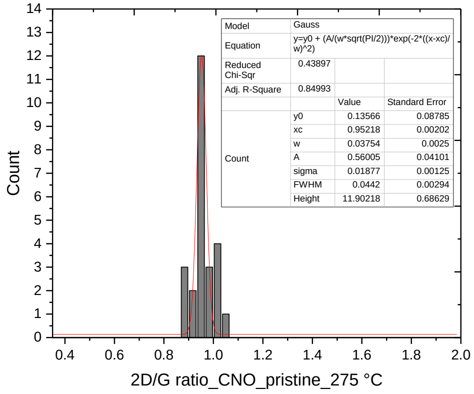
</td>
</tr>
</table>

325

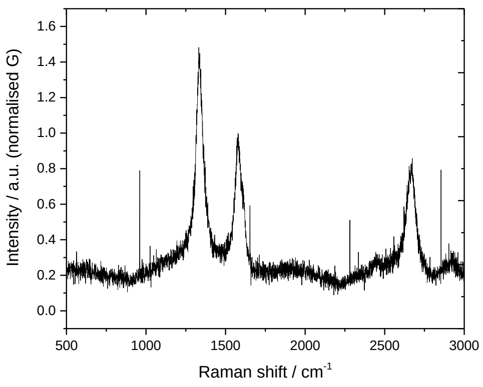
Intensity / a.u. (normalised G)
Raman shift / cm-1

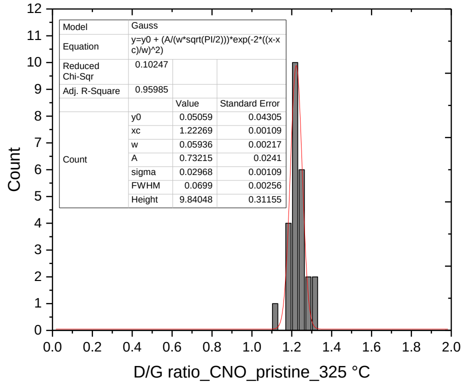
Model | Gauss
Equation | y=y0 + (A/(w*sqrt(PI/2)))*exp(-2*((x-xc)/w)^2)
Reduced Chi-Sqr | 0.10247
Adj. R-Square | 0.95985
Value | Standard Error
Count y0 | 0.05059 | 0.04305
xc | 1.22269 | 0.00109
w | 0.05936 | 0.00217
A | 0.73215 | 0.0241
sigma | 0.02968 | 0.00109
FWHM | 0.0699 | 0.00256
Height | 9.84048 | 0.31155
Count
D/G ratio_CNO_pristine_325 °C

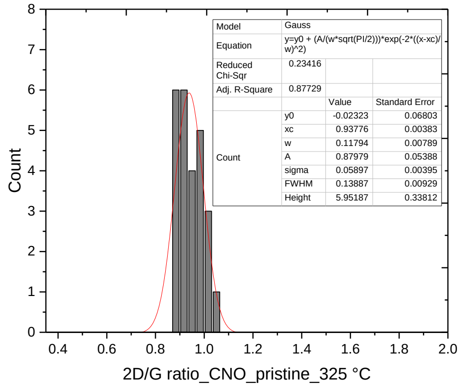
Model | Gauss
Equation | y=y0 + (A/(w*sqrt(PI/2)))*exp(-2*((x-xc)/w)^2)
Reduced Chi-Sqr | 0.23416
Adj. R-Square | 0.87729
Value | Standard Error
Count y0 | -0.02323 | 0.06803
xc | 0.93776 | 0.00383
w | 0.11794 | 0.00789
A | 0.87979 | 0.05388
sigma | 0.05897 | 0.00395
FWHM | 0.13887 | 0.00929
Height | 5.95187 | 0.33812
Count
2D/G ratio_CNO_pristine_325 °C

375

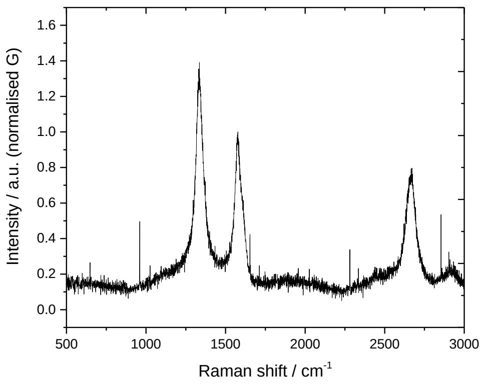
Intensity / a.u. (normalised G)
Raman shift / cm-1

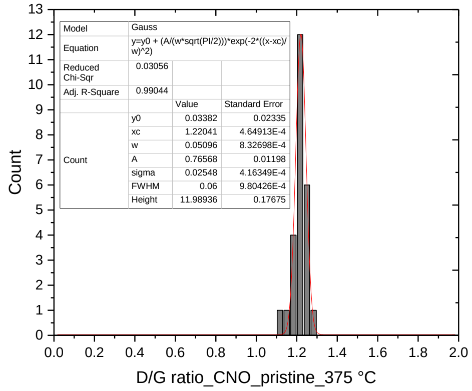
Model | Gauss
Equation | y=y0 + (A/(w*sqrt(PI/2)))*exp(-2*((x-xc)/w)^2)
Reduced Chi-Sqr | 0.03056
Adj. R-Square | 0.99044
Value | Standard Error
Count y0 | 0.03382 | 0.02335
xc | 1.22041 | 4.64913E-4
w | 0.05096 | 8.32698E-4
A | 0.76568 | 0.01198
sigma | 0.02548 | 4.16349E-4
FWHM | 0.06 | 9.80426E-4
Height | 11.98936 | 0.17675
Count
D/G ratio_CNO_pristine_375 °C

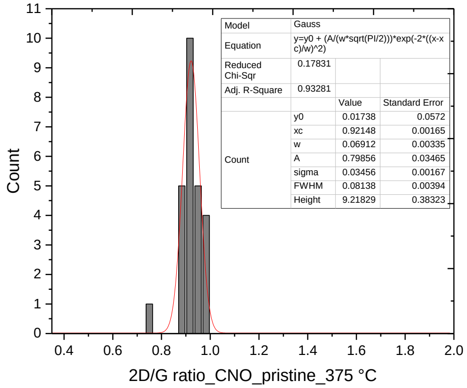
Model | Gauss
Equation | y=y0 + (A/(w*sqrt(PI/2)))*exp(-2*((x-xc)/w)^2)
Reduced Chi-Sqr | 0.17831
Adj. R-Square | 0.93281
Value | Standard Error
Count y0 | 0.01738 | 0.0572
xc | 0.92148 | 0.00165
w | 0.06912 | 0.00335
A | 0.79856 | 0.03465
sigma | 0.03456 | 0.00167
FWHM | 0.08138 | 0.00394
Height | 9.21829 | 0.38323
Count
2D/G ratio_CNO_pristine_375 °C

425	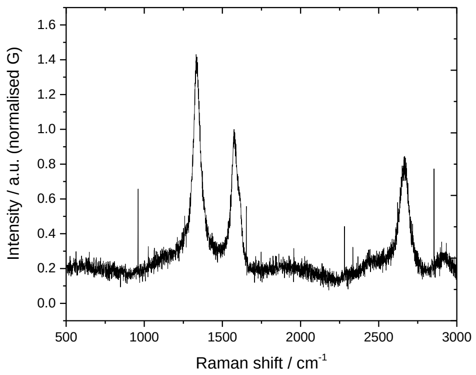	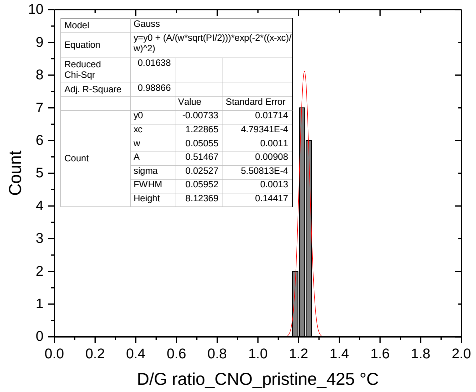	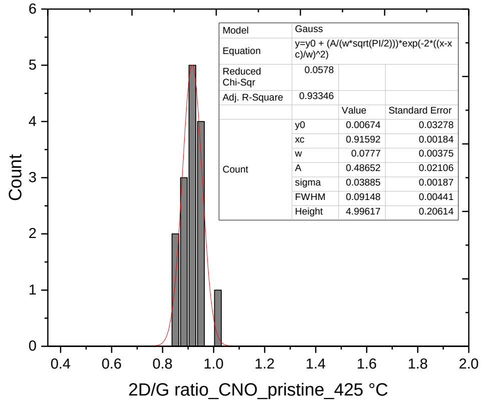

Intensity / a.u. (normalised G)
Raman shift / cm-1

Count
D/G ratio_CNO_pristine_425 °C
Model Gauss
Equation y=y0 + (A/(w*sqrt(PI/2)))*exp(-2*((x-xc)/w)^2)
Reduced Chi-Sqr 0.01638
Adj. R-Square 0.98866
Value Standard Error
y0 -0.00733 0.01714
xc 1.22865 4.79341E-4
w 0.05055 0.0011
A 0.51467 0.00908
sigma 0.02527 5.50813E-4
FWHM 0.05952 0.0013
Height 8.12369 0.14417

Count
2D/G ratio_CNO_pristine_425 °C
Model Gauss
Equation y=y0 + (A/(w*sqrt(PI/2)))*exp(-2*((x-xc)/w)^2)
Reduced Chi-Sqr 0.0578
Adj. R-Square 0.93346
Value Standard Error
y0 0.00674 0.03278
xc 0.91592 0.00184
w 0.0777 0.00375
A 0.48652 0.02106
sigma 0.03885 0.00187
FWHM 0.09148 0.00441
Height 4.99617 0.20614

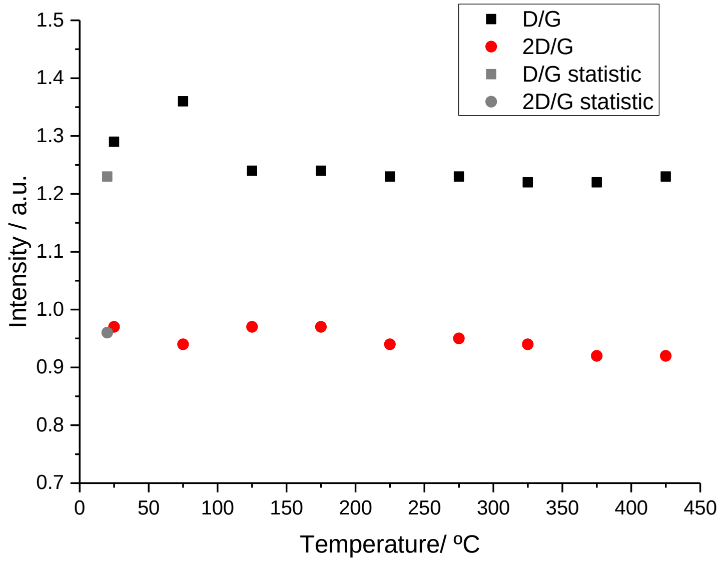

D/G
2D/G
D/G statistic
2D/G statistic
Intensity / a.u.
Temperature/ ºC

References

(1) Hotz, M. T.; Corbin, G. J.; Dellby, N.; Krivanek, O. L.; Mangier, C.; Meyer, J. C. Ultra-High Vacuum Aberration-Corrected STEM for in-Situ Studies. *Microsc Microanal* **2016**, *22* (S3), 34–35. https://doi.org/10.1017/S1431927616001021.

(2) E. Plonska-Brzezinska, M.; Echegoyen, L. Carbon Nano-Onions for Supercapacitor Electrodes: Recent Developments and Applications. *Journal of Materials Chemistry A* **2013**, *1* (44), 13703–13714. https://doi.org/10.1039/C3TA12628E.

(3) Molina-Ontoria, A.; Chaur, M. N.; Plonska-Brzezinska, M. E.; Echegoyen, L. Preparation and Characterization of Soluble Carbon Nano-Onions by Covalent Functionalization, Employing a Na–K Alloy. *Chem. Commun.* **2013**, *49* (24), 2406–2408. https://doi.org/10.1039/C3CC39077B.

(4) Bartelmess, J.; Luca, E. D.; Signorelli, A.; Baldrighi, M.; Becce, M.; Brescia, R.; Nardone, V.; Parisini, E.; Echegoyen, L.; Pompa, P. P.; Giordani, S. Boron Dipyrromethene (BODIPY) Functionalized Carbon Nano-Onions for High Resolution Cellular Imaging. *Nanoscale* **2014**, *6* (22), 13761–13769. https://doi.org/10.1039/C4NR04533E.

(5) Shenderova, O.; Grishko, V.; Cunningham, G.; Moseenkov, S.; McGuire, G.; Kuznetsov, V. Onion-like Carbon for Terahertz Electromagnetic Shielding. *Diamond and Related Materials* **2008**, *17* (4), 462–466. https://doi.org/10.1016/j.diamond.2007.08.023.

(6) Krueger, A.; Ozawa, M.; Jarre, G.; Liang, Y.; Stegk, J.; Lu, L. Deagglomeration and Functionalisation of Detonation Diamond. *physica status solidi (a)* **2007**, *204* (9), 2881–2887. https://doi.org/10.1002/pssa.200776330.

(7) Krüger, A.; Kataoka, F.; Ozawa, M.; Fujino, T.; Suzuki, Y.; Aleksenskii, A. E.; Vul', A. Ya.; Ōsawa, E. Unusually Tight Aggregation in Detonation Nanodiamond: Identification and Disintegration. *Carbon* **2005**, *43* (8), 1722–1730. https://doi.org/10.1016/j.carbon.2005.02.020.

(8) Zeiger, M.; Jäckel, N.; Aslan, M.; Weingarth, D.; Presser, V. Understanding Structure and Porosity of Nanodiamond-Derived Carbon Onions. *Carbon* **2015**, *84*, 584–598. https://doi.org/10.1016/j.carbon.2014.12.050.

(9) Reinert, L.; Zeiger, M.; Suárez, S.; Presser, V.; Mücklich, F. Dispersion Analysis of Carbon Nanotubes, Carbon Onions, and Nanodiamonds for Their Application as Reinforcement Phase in Nickel Metal Matrix Composites. *RSC Adv.* **2015**, *5* (115), 95149–95159. https://doi.org/10.1039/C5RA14310A.
